# Inhibition of Influenza A virus propagation by benzoselenoxanthenes stabilizing TMPRSS2 Gene G-quadruplex and hence down-regulating TMPRSS2 expression

**DOI:** 10.1038/s41598-020-64368-8

**Published:** 2020-05-06

**Authors:** Li-Wen Shen, Man-Qing Qian, Kai Yu, Suresh Narva, Fei Yu, Yan-Ling Wu, Wen Zhang

**Affiliations:** 1 Lab of Chemical Biology and Molecular Drug Design, College of Pharmaceutical Science, Zhejiang University of Technology, Hangzhou, 310014 China; 2 Institute of Drug Development & Chemical Biology, Zhejiang University of Technology, Hangzhou, 310014 China; 3 Lab of Chemical Biology, Medical School, Kunming University of Science and Technology, Kunming, 650500 China; 4 Lab of Molecular Immunology, Virus Inspection Department, Zhejiang Provincial Center for Disease Control and Prevention, Hangzhou, 310051 China

**Keywords:** Small molecules, Target identification, Influenza virus

## Abstract

Proteolytic cleavage of influenza A virus (IAV) hemagglutinin by host proteases is crucial for virus infectivity and spread. The transmembrane serine protease TMPRSS2 was previously identified as the essential protease that can cleave hemagglutinin of many subtypes of influenza virus and spike protein of coronavirus. Herein, we found that a guanine rich tract, capable of forming intramolecular G-quadruplex in the presence of potassium ions, in the promoter region of human TMPRSS2 gene was quite important for gene transcriptional activity, hence affecting its function. Furthermore, 7 new synthesized benzoselenoxanthene analogues were found to enable stabilizing such G-quadruplex. More importantly, compounds can down-regulate TMPRSS2 gene expression, especially endogenous TMPRSS2 protein levels, and consequently suppress influenza A virus propagation *in vitro*. Our results provide a new strategy for anti-influenza A virus infection by small molecules targeting the TMPRSS2 gene G-quadruplex and thus inhibiting TMPRSS2 expression, which is valuable for developing small molecule drugs against influenza A virus and also may be a potential candidate as anti- SARS-CoV-2 (Severe Acute Respiratory Syndrome CoV 2) lead molecules.

## Introduction

Influenza is an infectious viral infection that has serious impact on public health. Worldwide, annual influenza epidemics are estimated to result in about 3 to 5 million cases of severe illness, and about 290 000 to 650 000 respiratory deaths reported by the World Health Organization (WHO)^1^. Currently, the most widely used anti-influenza drugs are matrix protein 2 (M2) inhibitors amantadine, rimantadine and the neuraminidase (NA) inhibitors oseltamivir, zanamivir^2^. However, clinical applications of these drugs are limited since the rapid development of antiviral drug resistance. It was reported that virtually all 2009 pandemic H1N1 and seasonal H3N2 strains were resistant to M2 inhibitors, most of the seasonal H1N1 isolates were oseltamivir resistant^3^, and a few isolates resistant to oseltamivir even had appeared in 2013 Asian H7N9 strain^4^. The increasing frequencies of drug-resistant virus strain highlight the need for novel antiviral strategies, such as drugs focus on different viral targets or cellular factors.

Virus entry is the first and essential step in IAV life cycle, blocking of entry would lead to suppression of viral infectivity and consequently is an attractive antiviral strategy. Glycoprotein hemagglutinin (HA) of IAV plays a key role in the entry, since it confers virion ability of specific binding to host cells and subsequent membrane fusion. HA is synthesized as a fusion-inactive precursor (HA0) that could be cleaved into HA1 and HA2 fragments. The cleavage of HA0 is a necessary step for influenza virus to be contagious, as it liberates the fusion peptide which can insert into target membranes during fusion. On the other hand, the cleavage leads HA to be in a metastable conformation that can undergo the structural rearrangements required for fusion in endosome environment. However, IAV has no HA0 processing protease, cleavage of the HA0 is primarily accomplished by the host cellular proteases^5^.

Accumulating evidences suggest that, TMPRSS2, a type II transmembrane serine protease, is responsible for HA0 proteolytic cleavage of many subtype IAVs which contain a single arginine in the linker peptide between HA1 and HA2, such as H1N1 (A/PR/8/34) and H7N9 (A/Anhui/1/13)^6,7^. In animal models, TMPRSS2 deficiency protects mice against H1N1 (A/PR/8/34), mouse-adapted H1N1 (A/California/04/09) and H7N9 (A/Anhui/1/13) influenza A virus infections^6,7^. Genetic variants with higher TMPRSS2 expression confer higher risk to severe H1N1 (pdm09) and Asian H7N9 influenza A virus^8^. Furthermore, knocking out TMPRSS2 in mice does not cause any obvious phenotypic abnormality, such as death, infertility or visible sickness^9^. In addition, more recently, it is reported that TMPRSS2 can help SARS-CoV-2 for entry into host cells through entailing S protein cleavage^10^. The critical role in IAV and SARS-CoV-2 (Severe Acute Respiratory Syndrome CoV 2) infection and the nonvital physiological function of TMPRSS2 highlight an antiviral strategy that targets TMPRSS2 and inspire us to find small molecules that can inhibit IAV and SARS-CoV-2 infection via “chemical knockout” of TMPRSS2.

In the present study, we demonstrated that a guanine-rich tract of human TMPRSS2 promoter, located 90 to 118 nucleotide upstream of the transcription start site, was important for promoter activity and capable of forming intramolecular G-quadruplex, a noncanonical four-stranded DNA secondary structure that is comprised of two or more self-stack guanines quartets and was considered to be involved in regulation of gene transcription^11–13^. Next, we designed and synthesized a series of benzoselenoxanthene derivatives and found that these compounds can induce the formation of and stabilize the G-quadruplex structure in the guanine-rich tract, causing down-regulation of TMPRSS2 expression and finally resulted in a reduction of influenza A virus (A/PR8/34) titers *in vitro*. Our findings suggest that inhibiting TMPRSS2 expression by G-quadruplex stabilization in TMPRSS2 promoter is a potential strategy for IAV infection therapy and novel benzoselenoxanthene derivatives own a potential as drugs against IAV infection.

## Results

### The Guanine-rich tract is important for TMPRSS2 promoter activity

**Figure 1:**
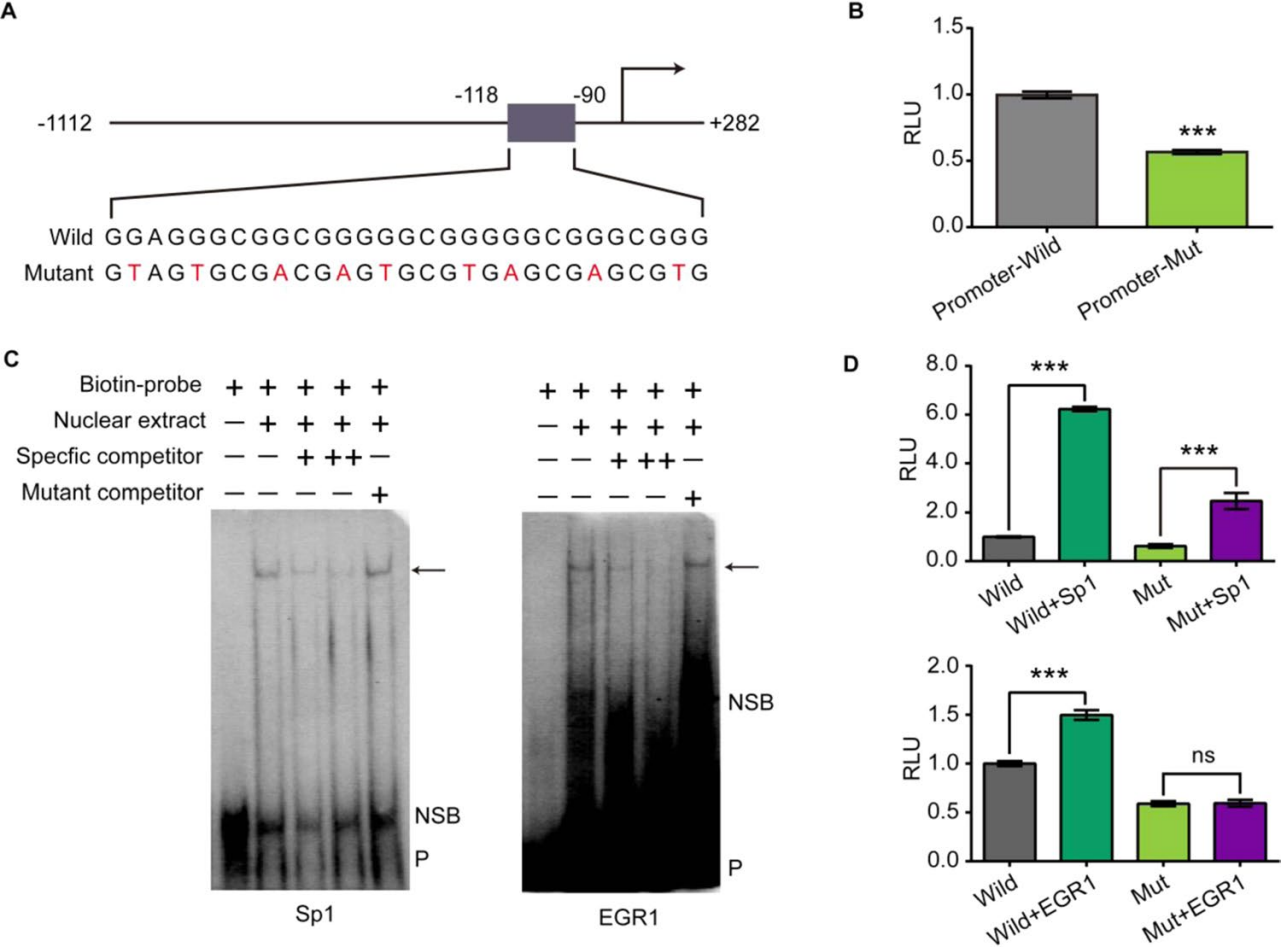
The guanine-rich tract (−118 to −90) is important for TMPRSS2 promoter activity. (**A**) Fragments inserted in pGL3-TMPRSS2-Wild and pGL3-TMPRSS2-Mut (mutations are indicated by red font). (**B**) Introducing mutations in the guanine-rich tract lead to a significant reduction of TMPRSS2 promoter activity (Supplementary Table S2). pGL3-TMPRSS2-Wild and pGL3-TMPRSS2-Mut were co-transfected with pRL-TK, luciferase expression was detected 24 h after transfection. (**C**) The guanine-rich tract contains Sp1 and EGR1 transcription factor binding site. Competition EMSA with Calu-3 nuclear extract. The DNA probes used for EMSA are listed in Supplementary Table S1. All probes are double stranded, but only the G-rich sequence is shown. Arrows: specific bind; NSB: nonspecific bind; P: free probe; -: not contain; +: contain; + (specific competitor): 50×specific competitor; ++ (specific competitor): 100×specific competitor; + (mutant competitor): 100×specific competitor. (**D**) Sp1 and EGR1 enhance wild but not mutant TMPRSS2 promoter activity (Supplementary Table S3). (Up) pCMV-Sp1 or pCMV-EGR1 was co-transfected with pGL3-TMPRSS2 and pRL-TK, luciferase expression was detected 24 h after transfection. (Down) pCMV-Sp1 or pCMV-EGR1 was co-transfected with pGL3-TMPRSS2-Mut and pRL-TK, luciferase expression was detected 24 h after transfection. All of experiments were repeated three times. Statistical significance was determined using Student’s two-tailed t-test (**B**,**D**). *P < 0.05, **P < 0.01, ***P < 0.001. Error bars represent the SEM of three independent experiments.

In order to investigate the role of the guanine-rich sequence in TMPRSS2 promoter activity, we performed luciferase assays using A549 cells. The reporter plasmid pGL3-TMPRSS2-Wild bears a wild-type TMPRSS2 promoter (−1112 to +282), while pGL3-TMPRSS2-Mut contains mutant in each guanine tract of the guanine-rich region (−118 to −90) (Fig. 1A). As shown in Fig. 1B, disrupting the guanine-tract resulted in 43% decline in luciferase expression. This implicates that the guanine-rich region is important for TMPRSS2 promoter activity.

Since the guanine-rich region was critical for TMPRSS2 promoter activity, we speculated that this tract contains some transcription factor bind site. To test the hypothesis, we searched for putative transcription factor binding site in this region with Jaspar (http://jaspardev.genereg.net/). Sp1 and EGR1 were identified as potential transcription factors that had multiple potential binding sites in the region. Then we confirmed the recruitment of Sp1 and EGR1 to this region by EMSA (Fig. 1C). The competition EMSA results demonstrated that the G-rich sequence indeed interacted with Sp1 and EGR1, and the protein: DNA complex was eliminated by the Sp1 and EGR1 consensus competitors but not the mutant competitors. These data provide preliminary evidence that the TMPRSS2 guanine-rich region contains Sp1 and EGR1 binding sites. In addition, when pCMV-Sp1 and pCMV-EGR1 were co-transfected with the pGL3-TMPRSS2-Wild, luciferase expression was increased by 5.2 folds and 0.5 folds, respectively. While we observed a 3-fold increase of luciferase expression when pCMV-Sp1 was co-transfected with pGL3-TMPRSS2-Mut and no significant luciferase expression changes when pCMV-EGR1 was co-transfected with pGL3-TMPRSS2-Mut (Fig. 1D). These results indicate that the guanine-rich region in TMPRSS2 promoter serves as Sp1 and EGR1 binding sequence and therefore is critical for TMPRSS2 transcription.

### The Guanine-rich region of the TMPRSS2 promoter forms intramolecular G-quadruplex structure

Previous research found that guanine-rich DNA in gene promoter can adopt G-quadruplex structure that played an important role in the regulation of gene transcription. To determine whether the guanine-rich sequence in TMPRSS2 promoter was also capable of forming an intramolecular/intermolecular G-quadruplex, we selected oligomeric DNAs (ODNs) corresponding to the region of interest, representing the sense, antisense strand or mutation sense strand (Supplementary Table S1). These ODNs were then used in electrophoretic mobility shift, UV-melting, circular dichroism (CD) and DMS footprinting studies.

**Figure 2:**
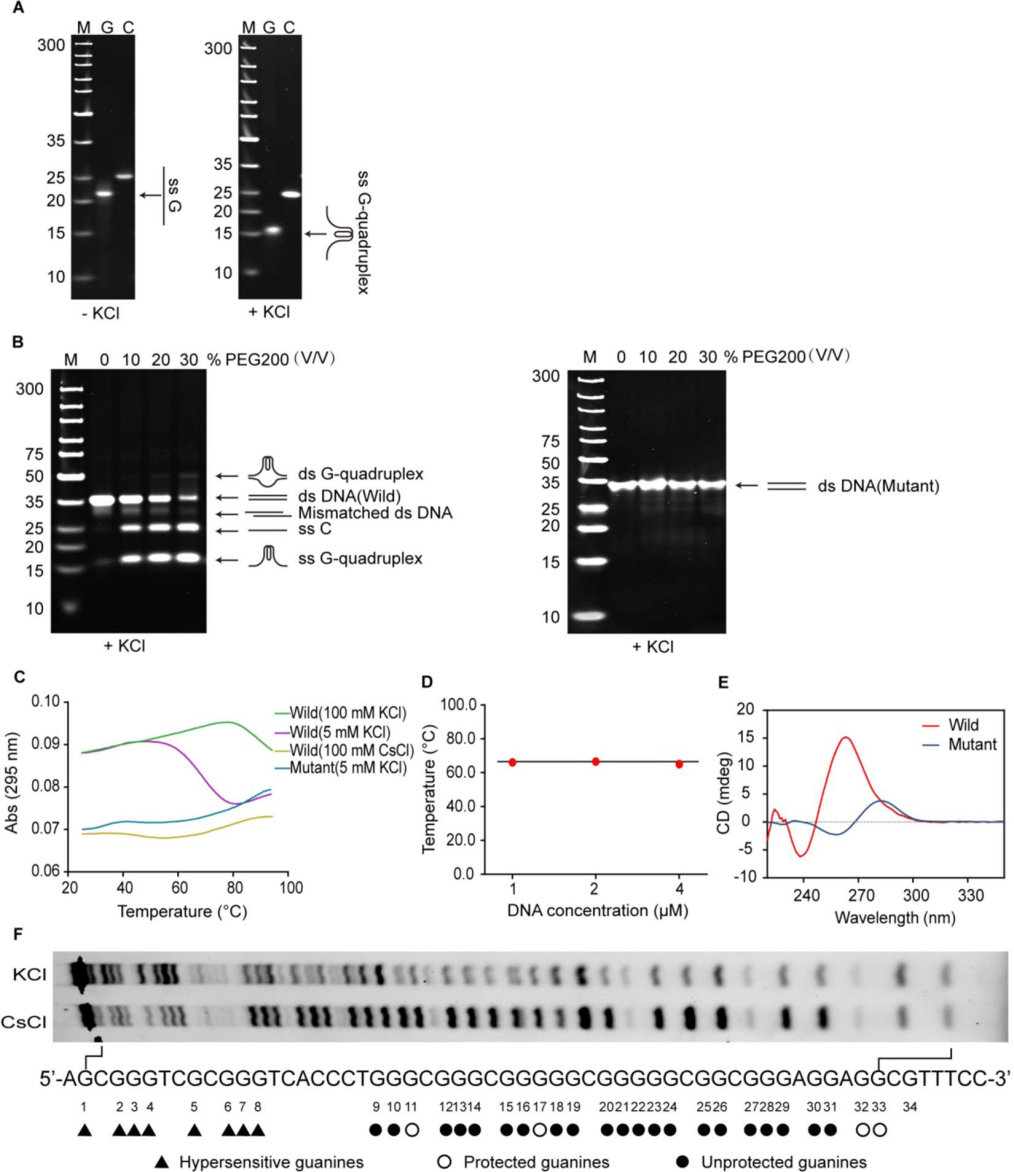
TMPRSS2-G adopts G-quadruplex structure in the presence of potassium ions. (**A**) TMPRSS2-G adopted G-quadruplex in the presence of KCl. Left: 1 μM TMPRSS2-G and TMPRSS2-C dissovled in Tris-HCl buffer (without KCl) respectively, after denaturation and renaturation, samples were subjected to native PAGE (without KCl); right: 1 μM TMPRSS2-G and TMPRSS2-C dissovled in Tris-HCl buffer (with 100 mM KCl) respectively, after denaturation and renaturation, samples were subjected to native PAGE (with 50 mM KCl). M: marker, G: TMPRSS2-G, C: TMPRSS2-C, ss G: linear free TMPRSS2-G, ss G-quadruplex: G-quadruplex formed by TMPRSS2-G. (**B**) PEG200 induces TMPRSS2 G-quadruplex structure formation in the presence of complementary strand (TMPRSS-C). 1 μM TMPRSS2-G + 1 μM TMPRSS2-C and 1 μM TMPRSS2-G-Mut + 1 μM TMPRSS2-C-Mut were incubated respectively with or without PEG200 to form complementary double-stranded DNA (ds DNA (Wild/Mutant)), after denaturation and renaturation, samples were subjected to native PAGE contained 50 mM KCl. ds G-quadruplex: complementary ds DNA contained G-quadruplex in the G-rich strand, ss C: TMPRSS2-C, ss G-quadruplex: G-quadruplex formed by TMPRSS2-G. (**C**) UV-melting curves of TMPRSS2-G resolved in Tris-HCl buffer (with 100 mM KCl, 5 mM KCl and 100 mM CsCl respectively). K^+^ but not Cs^+^ induces TMPRSS2-G to form G-quadruplex; TMPRSS2-G-Mut does not form G-quadruplex in the presence of KCl. (**D**) The *T*_*m*_ value of TMPRSS2-G is independent on DNA concentration. *T*_*m*_ values of 1 μM, 2 μM and 4 μM TMPRSS2-G resolved in Tris-HCl buffer (with 5 mM KCl) were obtained from UV-melting assays. (**E**) TMPRSS2-G exhibits characteristic CD spectra of parallel G-quadruplex. CD spectra of TMPRSS2-G and TMPRSS2-G-Mut resolved in Tris–HCl buffer (100 mM KCl) were collected at 25 °C. Three scans were averaged for each sample. Wild: TMPRSS2-G, mutant: TMPRSS2-G-Mut. (**F**) Guanines involved in G-quadruplex structure formation. KCl: TMPRSS2-G-FAM resolved in KCl contained buffer; CsCl: TMPRSS2-G-FAM resolved in CsCl contained buffer. All of experiments were repeated three times. Original images for A, B and F are shown in Supplementary Fig. S2.

It is generally considered that a guanine-rich oligomer folding into G-quadruplex structures is dependent on monovalent cations, such as potassium ions and sodium ions^14–18^. In electrophoretic mobility shift assay (EMSA), a DNA oligomer which can fold into an intramolecular G-quadruplex structure would migrate faster than a nonstructured single-stranded DNA, thus, it is possible to differentiate the intramolecular G-quadruplex structure from the other DNA structures by native PAGE^15^. As shown in Fig. 2A, the guanine-rich strand (TMPRSS2-G) dissolved in potassium-contained buffer migrated much faster than that dissolved in potassium-deficient buffer in native PAGE, suggesting that the TMPRSS2-G converted to the faster mobility species that might be intramolecular G-quadruplex in the presence of potassium ions; while the C-rich strand (TMPRSS2-C) dissolved in potassium-deficient buffer migrated as fast as that dissolved in potassium-contained buffer, indicating the TMPRSS2-C can’t form any secondary DNA under such experimental conditions. However, with the exception of telomeres, it is well known that DNA’s in genome mainly adopt the form of a right-handed double helix (B-DNA type). Because of the stability of the duplex DNA, there exists a barrier for the formation of G-quadruplex. It was reported that intracellular molecular crowded environment can promote the duplex DNA conversion to G-quadruplex configuration^16^. We, therefore, examined whether the desired TMPRSS2 G-quadruplex stably exists in the presence of complementary C-rich strand with or without PEG200 (a molecular crowding inducer) similar to actual *in vivo* crowded environment. In fact, the main band for complementary double-stranded DNA (ds NDA) migrated in line with 35 bp marker in the absence of PEG200, suggesting that it was insufficient to overcome the stability of the double-helix to form the G-quadruplex (Fig. 2B). While a new slower moving and weak band emerged corresponding to *ca*. 50 bp marker when two complementary strands were incubated in PEG200 contained buffer and the slower moving species could be explained due to formation of intramolecular G-quadruplexes in ds DNA. In addition, an ss C band from single-stranded C-rich TMPRSS2-C and a faster moving G-quadruplex band contributed to ss G-quadruplex formed by single-stranded G-rich TMPRSS2-G became stronger gradually with the increase of PEG200. These results indicate that such TMPRSS2 G-quadruplex structure exists in the presence of complementary C-rich strand or in ds DNA only under molecular crowded environment.

It was previously reported that G-quadruplex structures have a characteristic inverted melting curve at 295 nm^17^. In our study the G-quadruplex formation was further identified by UV-melting assay in the presence of potassium ions. As shown in Fig. 2C, UV-melting curve of TMPRSS2-G dissolved in K^+^-contained buffer was a distinct inverted S-type. However, when TMPRSS2-G dissolved in Cs^+^-contained buffer, the denaturation curve is flat. UV-melting assay results further reinforce G-quadruplex structure formation of TMPRSS2-G in the presence of potassium. Apart from these, *T*_*m*_ values of 1 μM, 2 μM and 4 μM TMPRSS2-G dissolved in K^+^-contained buffer were also measured respectively; the almost identical *T*_*m*_ values of different concentrations of oligonucleotide confirmed that such intramolecular G-quadruplex structure can be formed even in the presence of 5 mM K^+^ (Fig. 2D and Supplementary Fig. S1).

CD spectroscopy has been widely used for the characterization of G-quadruplex configuration. It was reported that parallel G-quadruplex had a positive peak at 260–265 nm and a trough at 240 nm, anti-parallel quadruplex exhibited a positive peak at 290–295 nm and a trough at 260–265 nm, whereas hybrid-type G-quadruplex had a trough at 240 nm and two positive peaks at 265–270 nm and 290–295 nm respectively^15^. Here, we acquired CD spectra of TMPRSS2-G and TMPRSS2-G-Mut in K^+^-contained buffer at 25 °C. The CD spectrum of TMPRSS2-G-Mut had a positive peak at 282 nm and a dip at 260 nm, which is characteristic of a single-stranded DNA. The TMPRSS2-G CD spectrum exhibited a characteristic positive peak at 264 nm and a dip at 240 nm, which is the typical CD signature of a parallel G-quadruplex structure (Fig. 2E). Therefore, CD spectroscopy suggests that the TMPRSS2-G sequence adopts parallel G-quadruplex in the presence of potassium ions.

Based on the above EMSA and CD results (Fig. 2B,C,E), we found that mutant single-stranded TMPRSS2-G (TMPRSS2-G-Mut) and double-stranded DNA (ds NDA (Mutant)) both cannot form G-qaudruplex under our experimental conditions.

In addition, by DMS footprinting assay, we further gain more additional information about guanines involved in G-quadruplex formation. DMS mediating methylation of guanine N7 would lead to DNA sensitivity to piperidine cleavage at sites occupied by guanines; however, the unique hoogsteen base pairing between guanines in a G-quadruplex structure protects the N7 position of guanine from DMS methylation, thus making DMS footprinting valuable for mapping G-quadruplex-forming regions^15^. The DMS footprinting results indicated that the N7 guanine methylation pattern produced by Cs^+^-contained sample was consistent with a single-stranded, unstructured form of DNA (Fig. 2F). While for the sample dissolved in K^+^-contained buffer, G9, G10, G12–16 and G18–31 showed protection or incomplete protection from modification by DMS, implying that these guanines participated in G-quadruplex formation. The complicated protection pattern suggested that the oligonucleotide might form several types of possible G-quadruplex structures and switch between these G-quadruplex structures.

### Benzoselenoxanthene derivatives stabilize TMPRSS2 G-quadruplex *in vitro*

**Figure 3:**
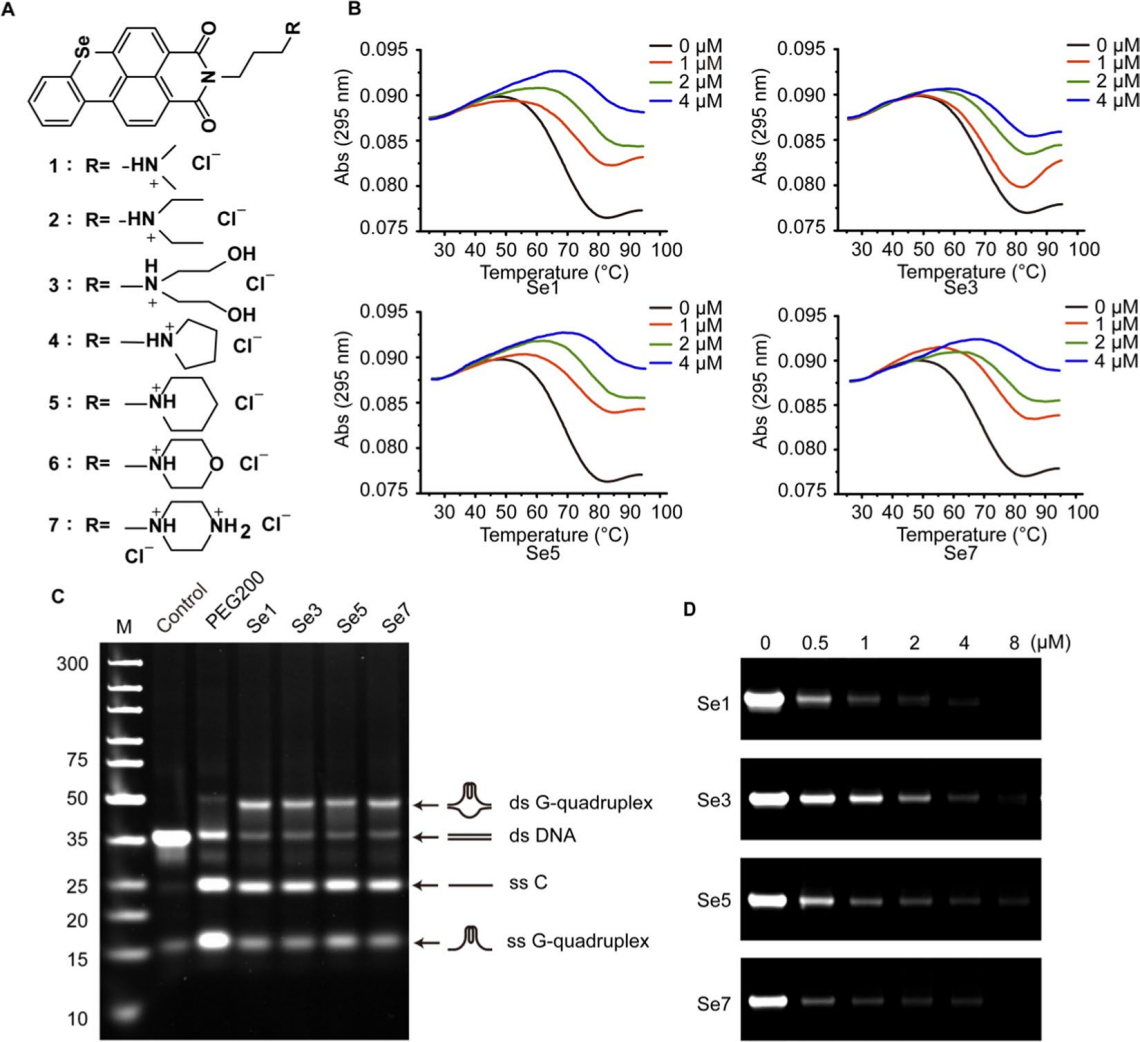
Benzoselenoxanthene analogues stabilize TMPRSS2 G-quadruplex in double-stranded TMPRSS2 DNA. (**A**) Chemical structures of benzoselenoxanthene analogues. (**B**) Benzoselenoxanthene analogues increase thermal stability of TMPRSS2 G-quadruplex. TMPRSS2-G was incubated with different concentrations of compounds in Tris-HCl buffer containing 5 mM KCl, then samples were denatured, renatured, and subjected to UV-melting assay. (**C**) Compounds stabilize TMPRSS2 G-quadruplex structure in the presence of complementary strand (TMPRSS-C) (ds G-quadruplex). TMPRSS2-G and TMPRSS2-C were incubated to form double-stranded TMPRSS2 DNA with or without (Control) 30% PEG200 or 8 μM compounds, then samples were denatured, renatured, and subjected to native PAGE in 50 mM K^+^-contained buffer. (**D**) Compounds inhibit the amplification of TMPRSS2 promoter region (cropped images). Compounds were added into the PCR systems and the PCR products were separated using 12% native PAGE. All of experiments were repeated three times. The original images for Figs. C, D are shown in Supplementary Fig. S3.

**Figure 4:**
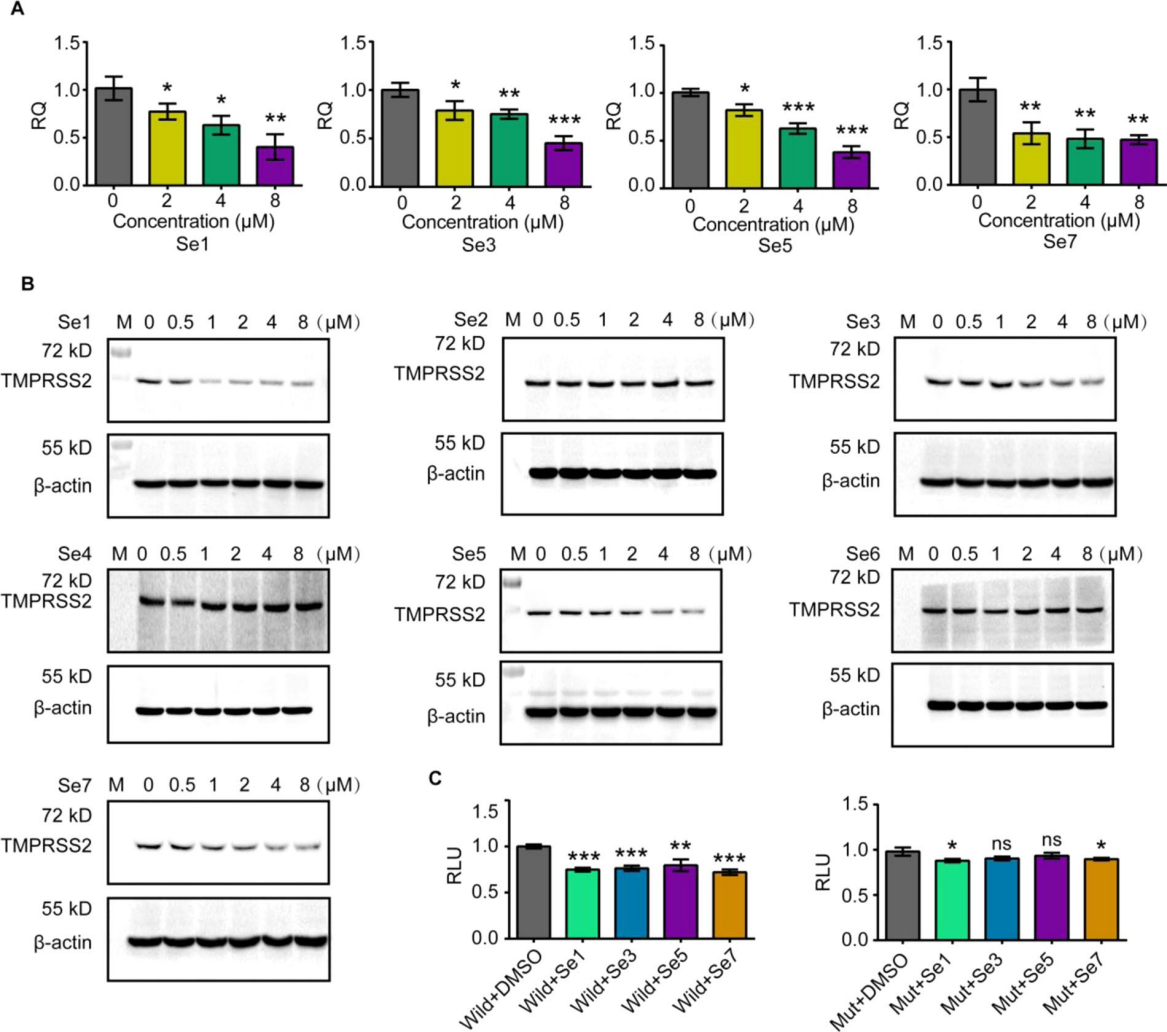
Benzoselenoxanthene analogues down-regulate TMPRSS2 expression through stabilizing TMPRSS2 G-quadruplex. (**A**) Compounds down-regulated the transcription of TMPRSS2 mRNA. After Calu-3 cells were treated with compounds, TMPRSS2 mRNA was examined by qPCR after 24 h. (**B**) Compounds down-regulated the expression of TMPRSS2 protein (Uncropped images shown in Fig. S4). After Calu-3 cells were treated with compounds, TMPRSS2 protein was examined by western blot after 24 h. (**C**) Compounds repressed wild or mutant TMPRSS2 promoter activity through stabilizing TMPRSS2 G-quadruplex. A549 cells were transfected with wild (left) and G-rich section mutant (right) TMPRSS2 promoter vector, then treated with 2 μM of compounds, finally luciferase was detected in 24 h after treatment with compounds. All of experiments were repeated three times. Statistical significance was determined using Student’s two-tailed t-test (**A**,**C**). *P < 0.05, **P < 0.01, ***P < 0.001. Error bars represent the SEM of three independent experiments.

We have demonstrated that TMPRSS2-G G-quadruplex formed in double-stranded DNA was insufficient to overcome the stability of the double-helix without molecular crowding environment similar to physiological conditions. In our previous study^18^, we found that benzothioxanthene analogues can induce the formation of and stabilize telomeric G-quadruplexes. To improve stability of the G-quadruplex structure in guanine-rich single strand and further stabilize the G-quadruplex structure in ds DNA, we designed and synthesized seven benzoselenoxanthene analogues (Fig. 3A) and examined the their ability to stabilize the G-quadruplex(es). However, we found that only four compounds Se**1**, Se**3**, Se**5** and Se**7** have effect on the expression of TMPRSS2 gene protein (see Fig. 4B). Thus, Four of them were chosen to test whether stabilization of G-quadruplex was achievable or not. According to UV melting assay results (Fig. 3B; Supplementary Table S4), it was clear that analogues Se**1**, Se**3**, Se**5** and Se**7** remarkably increased the *T*_*m*_ value of the G-quadruplex, indicating these compounds improved the stability of the TMPRSS2-G G-quadruplex. Further, by EMSA assay we examined if compounds could induce the formation of and stabilize G-quadruplex structures in ds DNA (TMPRSS2-G + TMPRSS2-C). As shown in Fig. 3C, a new slower moving and bright band and unwinding bands became stronger upon adding compounds relative to control. The slower moving band should be attributed to the ds G-quadruplex contained structure since it had a similar migration rate as the PEG200 induced ds G-quadruplex^16^. Results of UV melting and EMSA assay both confirmed that benzoselenoxanthene derivatives can stabilize the G-quadruplex structure and prevent it converting back to a double helix structure. In addition, PCR stop assay was used to detect whether the G-quadruplex stabilization would result in inhibition of replication or transcription or not. As expected, the strand extension catalyzed by Taq polymerse was inhibited and the final double-stranded PCR product was decreased along with the increase of drug concentration (Fig. 3D and Supplementary Fig. S3).

### Benzoselenoxanthene analogues down-regulate the expression of TMPRSS2 gene through stabilizing TMPRSS2 G-quadruplex structure

Giving the important role of the guanine-rich tract for TMPRSS2 promoter activity and the obvious stabilization effect of benzoselenoxanthene analogues on the TMPRSS2 G-quadruplex, we therefore speculated that these compounds could down-regulate TMPRSS2 expression as a consequence of stabilizing G-quadruplex. To test this hypothesis, the mRNA and protein expression of TMPRSS2 gene overexpressed in Calu-3 cells was measured in the presence of compounds by using quantitative real-time PCR and western blot analysis, respectively. As shown in Figs. 4A, 4B, Supplementary Fig. S4A, and Supplementary Table S5, the expression of TMPRSS2 gene decreased with increasing concentrations of compounds Se**1**, Se**3**, Se**5** and Se**7**. Of note, we observed that compounds Se**2**, Se**4**, and Se**6** almost have no influence on the expression of TMPRSS2 gene protein (Fig. 4B and Supplementary Fig. S4B) and the cause of the result is still unknown but under investigation. Thus, only four compounds Se**1**, Se**3**, Se**5** and Se**7** were used in other experiments. Next, dual luciferase assay was conducted to verify whether down-regulation of the gene expression resulted from G-quadruplex stabilization by compounds or not. As expected, in pGL3-TMPRSS2-Wild transfected group, luciferase expression was reduced by 25%, 24%, 20% and 28% upon the addition of 2 μΜ of compounds, Se**1**, Se**3**, Se**5** and Se**7**, respectively; while the pGL3-TMPRSS2-Mut driven luciferase activity was not affected so apparently by 2 μΜ of the compounds, Se**1**, Se**3**, Se**5** and Se**7**, with luciferase expression reduced by 12%, 10%, 7% and 10%, respectively (Fig. 4C; Supplementary Table S6). Collectively, these results strongly demonstrate that through stabilizing the G-quadruplex structure at cellular level, benzoselenoxanthene analogues can down-regulate the TMPRSS2 expression.

### Benzoselenoxanthene analogues inhibit influenza virus propagation *in vitro*

**Figure 5:**
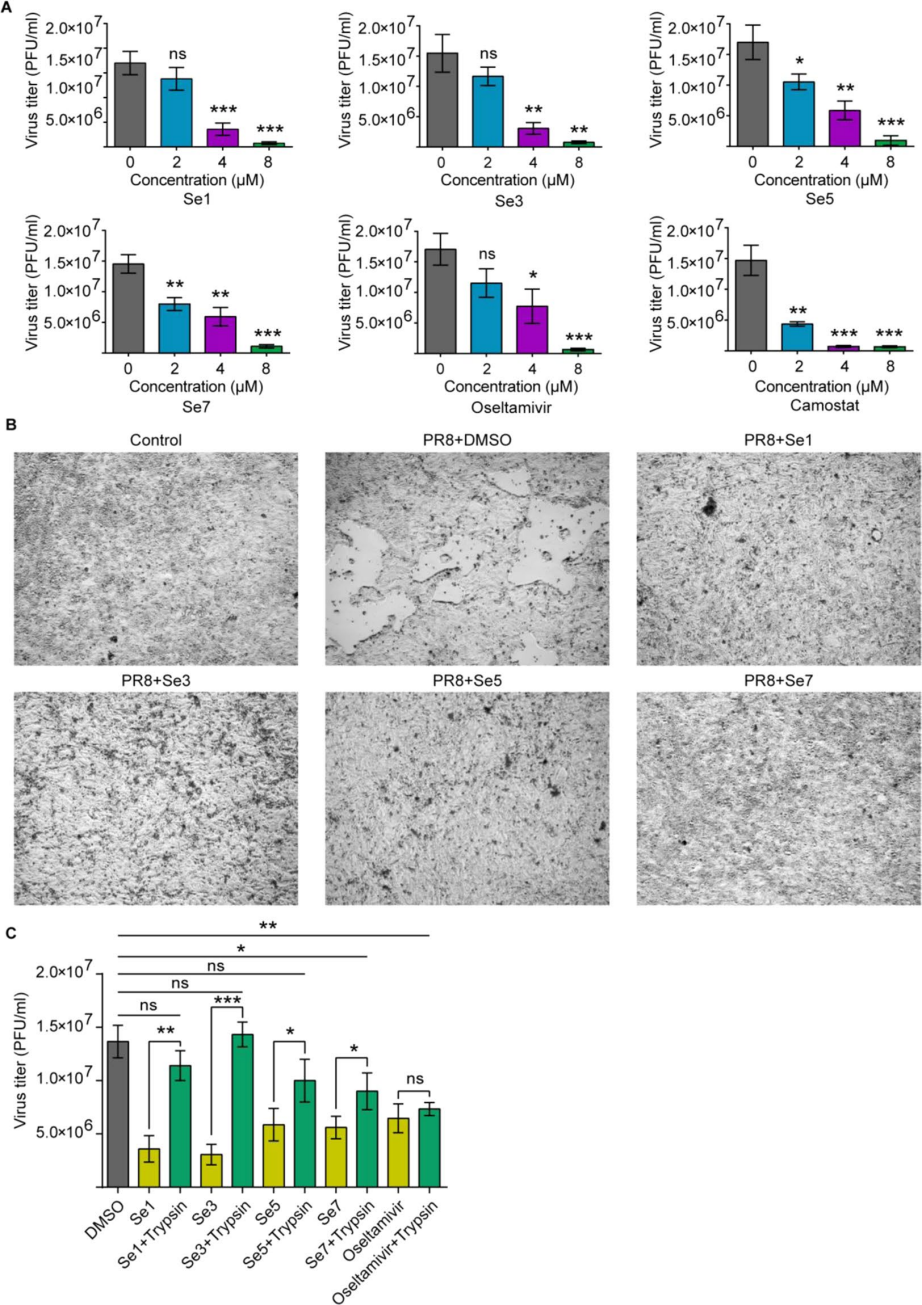
Benzoselenoxanthene analogues inhibit influenza virus propagation. (**A**) Compounds inhibit IAV (A/PR8/34) *in vitro*. Firstly, Calu-3 cells were treated with compounds or Oseltamivir and Camostat as references for 24 h, then, infected with IAV and followed by culture in compounds contained medium, finally, supernatants were collected at 48 h after infection and viral titers were determined by plaque assay. (**B**) Compounds suppressed cytopathic effect. After Calu-3 cells were treated with compounds (8 μM) and infected with IAV (A/PR8/34, herein, abbr.: PR8) as described above, photos were collected at 48 h after infection. (**C**) The inhibitory effects of Se1, Se3 and Se5 on IAV spread can be rescued by trypsin. Firstly, Calu-3 cells were treated with compounds or Oseltamivir as references for 24 h, then, infected with IAV and followed by culture in compounds and trypsin (4 μg/mL) contained medium, finally, supernatants were collected at 48 h after infection and viral titers were determined by plaque assay. All of experiments were repeated three times. Statistical significance was determined using Student’s two-tailed t-test (**A**,**C**). *P < 0.05, **P < 0.01, ***P < 0.001. Error bars represent the SEM of three independent experiments.

In light of the fact that benzoselenoxanthene analogues down-regulated TMPRSS2 expression through stabilizing TMPRSS2 G-quadruplex, we then explored whether this expression downregulation by compounds would inhibit IAV (A/PR8/34) replication *in vitro*. As seen in Fig. 5, Supplementary Fig. S5 and Supplementary Table S7, the viral growth was affected dramatically by all examined compounds in a roughly concentration dependent manner and the cytopathic effect (CPE) suppressed after treatment with compounds. In addition, we, also, examined that trypsin rescue the ability of novel designed compounds (Se**1**, S**3**, S**5** and Se**7**) and Oseltamivir as a positive drug to inhibit IAV infection. The results in Fig. 5C showed that the inhibitory effects of Se**1**, Se**3** and Se**5** can obviously be rescued by trypsin while that of oseltamivir cannot. This is because trypsin possesses the ability to cleave hemagglutinin (HA) of IAV, consequently facilitating IAV entry into host cells and this type of compounds target TMPRSS2 gene DNA sequence. While Oseltamivir as a neuraminidase inhibitor can efficiently block sialidase activity and significantly inhibit the releasing mechanism of the newly synthesized virion from the infected cell^19^. Taken together, it is expected that benzoselenoxanthene analogues could suppress IAV propagation in Calu-3 cells through down-regulating TMPRSS2 expression.

### Cytotoxicity assay

Cytotoxicity of Benzoselenoxanthene analogues was determined by a CCK-8 cell cytotoxicity assay. All examined compounds were found to be no significant cytotoxicity to Calu-3 at 10 μM of both benzoselenoxanthene and benzothioxanthene analogues and A549 cells at 1 μM of benzoselenoxanthene analogues in our studies (Supplementary Fig. S6).

## Discussion

Proteolytic cleavage of influenza A virus HA0 endows the virus fusion competency and therefore is critical for its infectivity. The redundant physiological functional host serine protease TMPRSS2 is hijacked by many subtypes influenza A virus and plays an important role in their HA-activating. In addition, TMPRSS2 also is involved in many other viral infections, such as coronavirus (SARS-CoV, MERS-CoV, HCoV-229E, hCoV-EMC) and hepatitis C virus (HCV)^20–24^. These suggest that TMPRSS2 is a promising target for viral infection therapy. Some protease inhibitors, such as Aprotinin and Camostat, exhibit excellent anti-influenza activity *in vitro*^25,26^. However, none of these inhibitors is TMPRSS2-specific. Their non-specific nature may lead to low bioavailability or unpredictable side effects. For instance, Aprotinin was found to increase the risks of myocardial infarction, heart failure, stroke and encephalopathy^27^.

In the present study, via a luciferase reporter assay, we found that a guanine-rich tract located within the region of TMPRSS2 promoter was important for its promoter activity. Competition EMSA assay revealed that repeat region contained bind sites of Sp1 and EGR1 transcription factor, suggesting the possibility of Sp1 and EGR1 were involved in TMPRSS2 transcription. Further co-transfect assay demonstrated that Sp1 and EGR1 promoted TMPRSS2 transcription through binding to the region. Some guanine-rich sequences existing in promoters of genes, such as BCL2, c-MYC, KRAS, VEGF and C-KIT, have been found being able to form G-quadruplex structure and thus regulate gene transcription, and therefore be regarded as targets for gene therapy^12^. By using native gel electrophoresis, thermal denaturing study and CD assay, we found the guanine-rich region in TMPRS2 promoter was also capable of forming parallel intramolecular G-quadruplex.

DMS footprinting assay provided additional information about guanine bases involved in G-quadruplex formation. However, the cleavage pattern was slightly indefinite because of no specific structure dominated. This likely was attributable to this sequence being able to form an equilibrium mixture of multiple intramolecular G-quadruplex structures. As the guanine rich sequence contained seven runs of at least two contiguous guanines, it was greatly possibly folded into multiple overlapping G-quadruplex structures. Such complexity was also found in the guanine-rich tracts of BCL2 P1 promoter and BCL2 major breakpoint region. It was speculated that the complexity of G-quadruplex formation is probably important to itself stability or differential binding of proteins^15,28,29^. In all, the complexity of the G-quadruplex region needs further more detailed studies.

Previously, we had identified that benzothioxanthene derivatives can stabilize telomeric G-quadruplex and thus inhibit multiple tumor cell lines growth^17^. Therefore we expected that those compounds could also stabilize TMPRSS2 G-quadruplex, down-regulate TMPRSS2 expression and suppress IAV propagation ultimately. Unfortunately, under our experimental conditions, they only stabilized TMPRSS2 G-quadruplex at extracellular molecular level (Supplementary Figs. S7 and S8), and cannot suppress TMPRSS2 transcription in the examined cell line (Supplementary Fig. S9). The conflicting results may be due to the lack of selective binding ability of compounds to TMPRSS2 G-quadruplex structure in cells. Recently, J. Seenisamy reported an Se-contained porphyrin, Se2SAP, which selectively bound to the c-MYC G-quadruplex in the presence of duplex DNA and other G-quadruplexes, implying that introduction of selenium made the compound less photoactive and noncytotoxic in comparison to TMPyP4^30^. Based on these studies, we designed and synthesized benzoselenoxanthene analogues. These compounds were found not only to greatly enhance the stability of the TMPRSS2 G-quadruplex structure at extracellular molecular level, but also to inhibit TMPRSS2 gene expression in Calu-3 cells. Moreover, more than 1 log10-unit reductions of virus titer were observed in cells treated with compounds, and the antiviral activity of these compounds were comparable to that of Oseltamivir.

In conclusion, our results provide direct evidence that G-quadruplex structure is formed in the guanine-rich sequence of TMPRSS2 promoter, and the gene expression could be regulated by ligand mediated G-quadruplex stabilization. Down-regulating TMPRSS2 expression by benzoselenoxanthene ananlogues significantly inhibits IAV growth and spread *in vitro*. These findings indicate that modulation of TMPRSS2 expression through G-quadruplex structure stabilization in the gene promoter may be a novel anti-influenza strategy and this strategy may lead to developing new small molecule drugs against IAV. Also, this is of important value for developing new drugs anti- SARS-CoV-2.

## Methods

### Chemical synthesis

**Figure 6:**
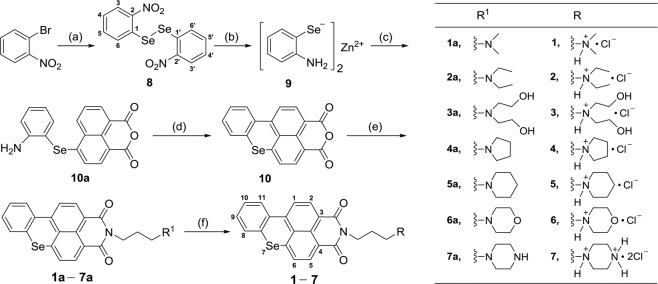
Reagents and Conditions. (**a)** Se, CuCl, 1,10-Phenanthroline, Cs_2_CO_3_, 1,4-dioxane, 120 °C, reflux, 20 h, 64% yield. (**b)** Zinc powder, CH_3_COOH, 75 °C, 15 min, 87% yield. (**c)** 4-Bromo-1,8-Naphthalic Anhydride, DMF, 75 °C, 2 h (without being separated). (**d)** Isoamyl nitrite, DMF, 85 °C, 2 h 87% yield. (**e)** R^1^(CH_2_)_3_NH_2_, Ethanol, 80 °C, reflux, 2 h, 85% yield. (**f)** HCl gas, 1–2 h, 75% yield.

The brief chemical synthesis steps were shown in Fig. 6. The detail synthesis, purification and characterisation of benzoselenoxanthene molecules are outlined in Supplementary Materials.

### DNA oligonucleotides

All oligonucleotides (Supplementary Table S1) used in the study were synthesized by Sangong, Inc. (Shanghai, China). Oligonucleotides were purified by HPLC-CE and dissolved in ddH_2_O to prepare 100 μΜ of DNA’s as stock solutions stored in −20 °C for use according to the manufacturer’s instruction

### Cells and influenza virus culture

Calu-3, A549 and MDCK cell lines were purchased from the Type Culture Collection of the Chinese Academy of Sciences, Shanghai, China. Calu-3 cells were cultured in DMEM/F12 media supplemented with 10% heat-inactivated fetal bovine serum, 1% Penicillin-Streptomycin, 1% MEM Non-Essential Amino Acids. A549 and MDCK cells were cultured in DMEM media supplemented with 10% heat-inactivated fetal bovine serum, 1% Penicillin-Streptomycin. Calu-3 and A549 have been authenticated by STR profiling (Supplementary Table S8). Mycoplasma contamination of all cell lines was tested every three months (Supplementary Table S9). H1N1 influenza A virus (A/PR8/34) was propagated in MDCK cells in serum-free DMEM medium contained 0.2% BSA and 2 μg/ml TPCK treated trypsin. Both cells and virus were cultured in a humidified atmosphere contained 5% CO_2_ at 37 °C.

### Dual luciferase assay

The DNA sequence of TMPRSS2 promoter (−1112 to +282 relative to the transcriptional start site) and mutant TMPRSS2 promoter containing mutations in guanine-rich region (−118 to −90) were synthesized by Sangon Inc (Shanghai, China). These fragments were subcloned into pGL3-basic vector. Sp1 and EGR1 expression vector (pCMV-Sp1 and pCMV-EGR1) were purchased from Sino Biological Inc (Beijing, China). For transient transfection experiments, A549 cells were seeded in 24-well dishes with a concentration of 5×10^4^ cells per well. Each well was co-transfected with 100 ng of pGL3-TMPRSS2-Wild or pGL3-TMPRSS2-Mut and 1 ng of pRL-TK with 8 uL Fugene HD. 48 h after transfection, the expression of firefly luciferase with respect to that of renilla luciferase was measured on SpectraMax M5 multimode reader. To confirm Sp1 and EGR1 transcription factor binding to guanine-rich region and to enhance promoter activity, A549 cells were co-transfected with 100 ng of pGL3-TMPRSS2-Wild or pGL3-TMPRSS2-Mut, 1 ng of pRL-TK and 100 ng of pCMV-Sp1 or pCMV-EGR1. The expression of firefly luciferase was determined as described above. To analyze the effect of benzoselenoxanthene analogues on TMPRSS2 promoter activity, A549 cells were transient transfected with pGL3-TMPRSS2-Wild plasmid or its mutant and pRL-TK, cells were treated with 2 μM of compounds at 6 h after transfection. The expression of firefly luciferase was determined at 18 h after treatment with compounds as described above.

### Competition EMSA

Competition electrophoresis mobility shift assays (EMSA) was performed according to the steps as following. Briefly, 5 μg of Calu-3 nuclear extract was incubated in 15 µl solution containing 1.5 µl 10× binding buffer, 1.0 µl Poly [dI: dC] for 20 min at RT; then to the mixture 0.5 µl Biotin-probe (with or without competitor) added and the mixture allowed to react at RT for 1 h; all the samples loaded in 6.5% polyacrylamide gel electrophoresis (PAGE), run on the gel on ice at 180 V, 1.5 h; transferred at 390 mA for 40 minutes; after transfer, the membrane placed in a UV linker to crosslink DNA; the membrane blocked with block buffer for 15 minutes on a shaker at RT; block buffer discarded and the membrane incubated with 15 mL of streptavidin-HRP bind buffer for 15 min on a shaker at RT. after block, the membrane was washed and 2.0 ml of ECL chemiluminescent substrates added onto the membrane, finally the chemiluminescent was detected by a chemiluminescence image.

### Native gel electrophoresis

1 μM TMPRSS2-G and 1 μM TMPRSS2-C were dissolved in 10 mM Tris-HCl buffer (pH 7.4, containing 100 mM KCl) respectively. The secondary structures of the DNA oligomers were obtained by heating samples at 95 °C for 10 min and then slowly cooling to room temperature over 4 h. After cooling to room temperature, samples were subjected to a 12% native PAGE in the presence of 50 mM KCl. As unstructured control, DNA oligomers were incubated in KCl-free Tris-HCl buffer. They were heated at 95 °C for 10 min then immediately put on ice before loading to a 12% native PAGE without KCl. To investigate that benzoselenoxanthene analogues can stabilize G-quadruplexes structure in the presence of a complementary strand, 1 μM TMPRSS2-G and 1 μM complementary strand (TMPRSS2-C) were dissolved in 10 mM Tris–HCl buffer (pH 7.4, containing 100 mM KCl) with or without compounds. They were heated at 95 °C for 10 min, then slowly cooling to room temperature. After cooling to room temperature, samples were subjected to a 12% native PAGE in the presence of 50 mM KCl. After electrophoresis, gels were stained with SYBR Gold and analyzed by imaging analyzer.

### UV melting assay

1 μM TMPRSS2-G was denaturated at 95 °C in 10 mM Tris-HCl buffer (pH 7.4, containing KCl or CsCl) for 10 min then slowly cooling to room temperature. samples were then subjected to UV melting analysis. The UV melting assay was performed on a Shimadzu UV-2550 spectrophotometer equipped with a digital circulating water bath. The temperature was scanned from 25 °C to 95 °C at a heating rate of ~0.5 °C/min. To study the G-quadruplex thermodynamic stability by benzoselenoxanthene analogues, solutions containing 1 μM TMPRSS2-G and different concentrations of compounds were prepared in 10 mM Tris-HCl buffer (pH 7.4, containing 5 mM KCl) and subjected to UV melting analysis as described above.

### Circular Dichroism

Circular dichroism (CD) experiments were carried out with 2 μM TMPRSS2-G or TMPRSS2-G-Mut DNA dissolved in 10 mM Tris-HCl buffer (pH 7.4, containing 100 mM KCl). Samples were denatured at 95 °C and slowly cooled to room temperature for secondary structure formation. Spectra was recorded in 5 mm path length quartz cuvette at a scanning rate of 100 nm/min over a wavelength range of 200–350 nm by CD spectropolarimeter (J-810, Jasco, Japan). Three scans were averaged for each sample and baseline was corrected according to blank buffer spectra.

### Dimethyl sulfate footprinting

200 μL TMPRSS2-G-FAM (0.1 μM) in 10 mM Tris-HCl buffer (pH = 7.4, containing 100 mM KCl or CsCl) was denatured by heating at 95 °C for 10 min and then slowly cooled down to room temperature. After cooling to room temperature, samples were treated with 4 μL of 10% dimethyl sulfate (DMS) at 25 °C for 10 min. The reaction was then terminated with 200 μL stop solution on ice (1.5 M sodium acetate, pH7.0, 1 M β-mercaptoethanol, 1 mg/mL yeast tRNA), followed by phenol/chloroform extraction and ethanol precipitation. After ethanol precipitation and washing, the cleavage reaction was performed by heating the samples at 90 °C for 30 min in 100 μL of 10% piperidine. Cleavage reaction was then stopped by chilling in ice, followed by 300 μL chloroform extraction twice and precipitation with 0.1 volume of sodium acetate (3 M, pH = 5.2) and 3 volumes of ethanol. Finally, The DNA samples were resuspended in 50% formamide, denatured at 95 °C and then analyzed on 16% denatured PAGE.

### PCR stop assay

PCR reaction system contained 1 μM of TMPRSS2-L and TMPRSS2-Rev, 0.2 mM dNTP, 0.1 U/μL taq polymerase and different concentrations of compounds. PCR setting was as follow: 94 °C for 5 min, followed by 10 cycles of 94 °C for 30 s, 60 °C for 30 s, and 72 °C for 30 s. Amplified products were run on 12% native PAGE and stained by SYBR Gold.

### Cytotoxicity assay

Cells were seeded in 96-well plates, after growing to 90% confluence, treated with DMSO (1% total volume) or benzoselenoxanthene compounds for 48 h. Then 10 μL of CCK-8 stock solution was added to each well, after 1 h incubation, the absorbance at 450 nm was measured on SpectraMax M5 multimode reader. DMSO (control) was defined as 100% cell viability.

### Real Time PCR

Calu-3 cells were seeded into 6 well culture plate, after growing to 90% confluence, cells were treated with benzoselenoxanthene compounds for 24 h. RNA was extracted with TRIzol reagent. 500 ng of total RNA was used in a 20 μL cDNA synthesis reaction using ReverTra Ace qPCR RT-Kit (TOYOBO, FSK-100) according to the manufacturer’s protocol. For quantification of mRNA levels, 10 μL of reaction mixture containing 1 μL of the cDNA, 5 μL SYBR Green Mix, 0.5 μM each of forward and reverse primers was used for quantification on a real-time PCR apparatus (ABI StepOnePlus). The program was consisted of a pre-denaturing cycle of 10 min at 95 °C, 40 cycles of PCR (95 °C for 15 s, 60 °C for 15 s, and 72 °C for 45 s). Three times PCR replications were performed and the transcription levels were normalized to GAPDH by using the 2^−ΔΔCt^ method.

### Western blot analysis

Calu-3 cells were seeded in 6 well culture plate, after growing to 90% confluence, treated cells with benzoselenoxanthene compounds for 24 h. Cells from each well were then lysed by 150 μL RIPA lysis buffer for 10 min on ice. Cell lysates were collected and then cleared by centrifugation at 15000 g at 4 °C for 10 min. Protein concentration was determined by BCA protein assay. For western blot analysis, 15 μg protein was resuspended in loading buffer and heated at 100 °C for 10 min. After denaturalization, pellets were loaded onto 12% polyacrylamide gel, followed by transferring to a PVDF membrane and incubated with specific antibodies, TMPRSS2 antibody (abcam, ab92323), β-actin antibody (abcam, ab8827) and anti-Rabbit IgG H&L (HRP) (abcam, ab6721)^31–33^. Protein band was stained with ECL substrate and visualized in a chemiluminescence imager system.

### Antiviral activity

To investigate the antiviral activity of benzoselenoxanthene analogues, Calu-3 cells were grown to confluence in 6 well plate, treated with compounds for 24 h, then challenged with influenza A virus (A/PR8/34) at a MOI of 0.0001, followed by washing, replenishing with fresh serum-free DMEM medium containing compounds. After another 48 h, supernatants were collected and viral titers were determined by plaque assay as described in other research^34^. The virus titer was presented as plaque-forming units per ml (pfu/ml).

### Quantification and statistical analysis

All of real time RT-PCR assays, dual luciferase assays, cytotoxicity assays and antiviral activity experiments were repeated three times. Data obtained from these experiments were statistically analyzed by using unpaired two-tailed Student’s t-test. All values in the text and figure legends were represented as the mean ± SEM. P  <  0.05 (*p  <  0.05, **p  <  0.01, ***p  <  0.001) was considered to indicate significant difference.

## Supplementary information

Supplementary information.

## Data Availability

All data generated or analysed during this study are included in this published article and its Supplementary Information files.
