# Supplementary material for: Inhibition of Influenza A virus propagation by benzoselenoxanthenes stabilizing TMPRSS2 Gene G-quadruplex and hence down-regulating TMPRSS2 expression
Source: Sci Rep. 2020 May 6;10:7635. doi: 10.1038/s41598-020-64368-8 (PMC7203196; doi:10.1038/s41598-020-64368-8)
Supplement: Supplementary file 1 — Supplementary information. [file 41598_2020_64368_MOESM1_ESM.docx]

**Supplementary information**

**Inhibition of Influenza A virus propagation by benzoselenoxanthenes stabilizing TMPRSS2 Gene G-quadruplex and hence down-regulating TMPRSS2 expression**

Li-Wen Shen^1,2^, Man-Qing Qian^1,2^, Kai Yu^1,2^, Narva Suresh^1,2^, Fei Yu^3^, Yan-Ling Wu^4^*, & Wen Zhang^1,2^*

**The detailed information for synthesis of compounds**

**General**

Seven novel benzo[k,l] selenoxanthene-3,4-dicarboximide hydrochloride Se**1**-Se**7** were synthesized according to the established method by our Lab in Fig. 6. Melting points were taken on a XT-5A micro-melting point measure apparatus made in Shanghai and uncorrected. Reagents and solvents with high quality were purchased from standard suppliers and used without further purification. Reaction processes were tracked by TLC on silica gel-precoated F254 Merck plates and the thin layer plates were examined under the UV lamps (254 nm and 365 nm). NMR spectra were recorded with AVANCE III 500 MHz nuclear magnetic resonance spectrometer (Bruker, Switzerland). Chemical shift (δ) were reported in ppm and coupling constants (J) in herts. The following abbreviations were used to explain multiplicities: s, singlet; d, doublet; t, triplet; q, quartet; m, multiplet. ESI-TOF-Mass spectrometry was produced on an Agilent 6210 TOF LC/MS spectrometer (Agilent, America). Elemental composition was analysized on a CarloErba1106 Analyser.

**General procedure for the synthesis of benzo[*k*, *l*]selenoxanthene-3,4-dicarboximide hydrochloride and their intermediates and characterization of compounds**

(a) 1-Bromo-2-nitrobenzene 5.00 g (24.8 mmol), elemental selenium powder 5.86 g (74.2 mmol), CuCl 1.23 g (12.4 mmol), Cs_2_CO_3_ 24.2 g (74.3 mmol), and 1,10-phenanthroline 2.45 g (12.4mmol), and 1, 4-dioxane (50 mL) were introduced into a 100 mL three-necked round-bottomed flask equipped with a magnetic stir bar, and the resulting mixture was stirred under N_2_ atmosphere at 120 °C for 24 h till starting material was reacted completely by TLC. Then the solution was cooled to room temperature and filtered by the diatomite to remove excess selenium powder, and the filtrate was concentrated by a rotary evaporator. The residue was purified by column chromatography on silica gel using petroleum ether: etheylacetate (3:1) as an eluent to give 3.19 g (yield: 64.0%) of **8**: brown solid; mp: 210‒212 °C (lit. 206‒208 °C). ^1^H NMR (500 MHz, DMSO-*d_6_*): δ8.40 (dd, *J* = 8.2, 1.3 Hz, 2H, 3-H, 3’-H), 7.89 (dd, *J* = 8.1, 1.1 Hz, 2H, 6-H, 6’-H), 7.74-7.69 (m, 2H, 5-H, 5’-H), 7.62-7.57 (m, 2H, 4-H, 4’-H). MS-EI (m/z): 358[(M-NO_2_)^+^], 312[(M-2NO_2_)^+^], 251[(M-C_6_H_4_N_2_O_3_)^+^].

(b) Zinc dust was added gradually to a gently boiling solution of bis(2-nitrophenyl) diselenide (3.19 g) in 100 mL of glacial acetic acid, until the color of the solution was discharged, which occurred usually in about fifteen minutes. The boiling solution was filtered rapidly, by means of a Buchner funnel, into a large volume of water, and the zinc salt then precipitated in crystalline form. It was washed firstly with water, then with boiling alcohol and dried to give 2.81 g (yield: 87.0%) of **9**: pale white solid; mp: 248‒249 °C (lit. 253 °C).

(c) A mixture of 2.81 g (6.90 mmol) of **9** and 3.82 g (13.8 mmol) of 4-bromo-1, 8-naphthalimides was dissolved in 60 mL of DMF, and the resulting mixture was stirred under N_2_ atmosphere at 75 °C for 2 h to afford **10a**.

(d) The formed anhydride **10a** was not separated and directly cyclized by the addition of 2.63 mL (38.64 mmol) of isoamyl nitrite under N_2_ atmosphere at 85 °C for 2 h. After removal of solvent, the residues was separated by silica gel chromatography using petroleum ether:ethyl acetate (5:1) as an eluent to obtain 4.15 g of benzoselenoxanthene-3,4-dicarboxylic anhydride **10** (yield: 69%): pure orange solid, mp: 290‒292 °C, 96.5% purity by HPLC. ^1^H NMR (500 MHz, DMSO-*d_6_*): δ 8.53 (m, 3H, 1-H, 2-H, 5-H), 8.26 (m, 1H, 6-H), 8.07 (m, 1H, 8-H), 7.79 (m, 1H, 11-H), 7.50 (m, 2H, 9-H, 10-H). MS-EI: calculated m/z for C_18_H_8_O_3_Se: 351.96; found: 352 [M]^+^, 308 [(M-CO_2_)^+^], 280 [(M-C_2_O_3_)^+^].

(e) Benzoselenoxanthene-3,4-dicarboxylic anhydride **10** was suspended in ethanol and an excess of amine or diamine was added. The liquor was stirred under reflux conditions for 2 h. After evaporation of the solvent, the naphthalimides were isolated by column chromatography over SiO_2_ (CH_2_Cl_2_: MeOH = 20:1, v/v) to afford compounds **1a‒7a**.

(f) Compounds **1a‒7a** were dissolved in trichloromethane, and then resultant solution was stirred the under room temperature, meanwhile HCl gas put through it for 2 h. After evaporation of the solvent, the hydrochlorides **1‒7** were purified on silica gel (CH_2_Cl_2_: MeOH =15:1, v/v).

Data of characterization for the precusors **1a‒7a** and targets **1‒7** as follows:

N-(3-(dimethylamino)propyl)benzo[*k*, *l*] selenoxanthene-3,4-dicarboximide **1a**:

R_f_: 0.22 (DCM/methanol 20:1), yellow solid, yield: 92%, mp:184‒186 °C, 95.1% purity by HPLC. ^1^H NMR (500 MHz, CDCl_3_): δ 8.52 (d, *J* = 8.1 Hz, 1H, 5-H), 8.28 (d, *J* = 7.8 Hz, 1H, 2-H), 8.19 (d, *J* = 8.2 Hz, 1H, 1-H), 8.14 (d, *J* = 8.1 Hz, 1H, 6-H), 7.64 (d, *J* = 7.8 Hz, 1H, 8-H), 7.45(d, *J* = 7.5 Hz, 1H, 11-H), 7.39 (t, *J* = 7.2 Hz, 1H, 9-H), 7.34 (t, *J* = 7.3 Hz, 1H, 10-H), 4.25-4.18 (m, 2H, CONCH_2_), 2.58 (t, *J* = 7.4 Hz, 2H, CH_2_N^+^), 2.37 (s, 6H, N(CH_3_)_2_), 2.06-1.95 (m, 2H, NCH_2_*CH_2_*CH_2_N).

N-(3-(diethylamino)propyl)benzo[*k*, *l*] selenoxanthene-3,4-dicarboximide **2a**:

R_f_: 0.21 (DCM/methanol 20:1), yellow solid, yield: 92%, mp: 154‒156 °C, 96.0% purity by HPLC. ^1^H NMR (500 MHz, CDCl_3_): δ 8.52 (d, *J* = 8.2 Hz, 1H, 5-H), 8.28 (d, *J* = 7.8 Hz, 1H, 2-H), 8.18 (d, *J* = 8.3 Hz, 1H, 1-H), 8.14 (d, *J* = 7.9 Hz, 1H, 6-H), 7.63 (t, *J* = 6.8 Hz, 1H, 8-H), 7.48-7.42 (dd, *J* = 1.2, 7.6 Hz, 1H, 11-H), 7.42-7.36 (m, 1H, 9-H), 7.36-7.30 (m, 1H, 10-H), 4.24-4.15 (m, 2H, CONCH_2_), 2.78-2.62 (m, 6H, 2×^+^NCH_2_, CH_2_N^+^), 1.98 (m, 2H, NCH_2_*CH_2_*CH_2_N), 1.15-1.05 (m, 6H, 2×CH_3_).

N-(3-(Diethanolamino)propyl)benzo[*k*, *l*] selenoxanthene-3,4-dicarboximide **3a**:

R_f_: 0.19 (DCM/methanol 15:1), orange yellow solid, yield: 92%, mp: 102‒104 °C, 97.8% purity by HPLC. ^1^H NMR (500 MHz, DMSO-*d_6_*): δ 8.28-8.17 (m, 3H, 1-H, 2-H, 5-H), 8.02 (d, *J* = 7.8 Hz, 1H, 6-H), 7.72 (d, *J* = 7.8 Hz, 1H, 8-H), 7.60-7.55 (m, 1H, 11-H), 7.40-7.33 (m, 2H, 9-H, 10-H), 3.99-3.91 (m, 2H, CONCH_2_), 3.47 (t, *J* = 6.3 Hz, 4H, 2×C*H_2_*OH), 2.68-2.54 (m, 6H, 2×^+^NCH_2_, CH_2_N^+^), 1.81-1.71 (m, 2H, NCH_2_*CH_2_*CH_2_N).

N-(3-(pyrrolidin-1-yl)propyl)benzo[*k*, *l*] selenoxanthene-3,4-dicarboximide **4a**:

R_f_: 0.20 (DCM/methanol 20/1, v/v), yellow solid, yield: 92%, mp: 215‒217 °C, 96.3% purity by HPLC. ^1^H NMR (500 MHz, DMSO-*d_6_*): δ 8.33 (d, *J* = 8.5 Hz, 1H, 5-H), 8.30-8.24 (m, 2H, 1-H, 2-H), 8.07 (d, *J* = 7.8 Hz, 1H, 6-H), 7.80 (d, *J* = 7.8 Hz, 1H, 8-H), 7.67-7.61 (m, 1H, 11-H), 7.43-7.38 (m, 2H, 9-H, 10-H), 4.04 (t, *J* = 6.7 Hz, 2H, CONCH_2_), 3.50 (m, 2H, ^+^NCH_2_), 3.23-3.18 (m, 2H, ^+^NCH_2_), 2.96 (m, 2H, CH_2_N^+^), 2.12-2.04 (m, 2H, NCH_2_*CH_2_*CH_2_N), 2.01-1.83 (m, 4H, 2 × CH_2_ (cyclo)).

N-(3-(piperidin-1-yl)propyl)benzo[*k*, *l*] selenoxanthene-3,4-dicarboximide **5a**:

R_f_: 0.23 (DCM/methanol 20/1, v/v), yellow solid, yield: 92%, mp: 157‒159 °C, 97.2% purity by HPLC. ^1^H NMR (500 MHz, CDCl_3_): δ 8.48 (d, *J* = 8.1 Hz, 1H, 5-H), 8.25 (d, *J* = 7.8 Hz, 1H, 2-H), 8.13 (dd, *J* = 14.9, 8.2 Hz, 2H, 1-H, 6-H), 7.60 (d, *J* = 7.8 Hz, 1H, 8-H), 7.43 (d, *J* = 7.5 Hz, 1H, 11-H), 7.40-7.35 (m, 1H, 9-H), 7.35-7.31 (m, 1H, 10-H), 4.20 (t, *J* = 7.2 Hz, 2H,CONCH_2_), 2.71-2.48 (m, 6H, 2×^+^NCH_2_, CH_2_N^+^), 2.11-2.00 (m, 2H, NCH_2_*CH_2_*CH_2_N), 1.66 (m, 4H, 2×CH_2_ (cyclo)), 1.45 (m, 2H, CH_2_*CH_2_*CH_2_(cyclo)).

N-(3-morpholinopropyl)benzo[*k*, *l*] selenoxanthene-3,4-dicarboximide **6a**:

R_f_: 0.20 (DCM/methanol 20/1, v/v), yellow solid, yield: 92%, mp: 131‒133 °C, 98.4% purity by HPLC. ^1^H NMR (500 MHz, CDCl_3_): δ 8.51 (d, *J* = 8.2 Hz, 1H, 5-H), 8.27 (d, *J* = 7.8 Hz, 1H, 2-H), 8.17 (t, *J* = 8.0 Hz, 1H, 1-H), 8.14 (d, *J* = 7.8 Hz, 1H, 6-H), 7.66-7.61 (m, 1H, 8-H), 7.44 (dd, *J* = 7.6, 1.3 Hz, 1H, 11-H), 7.41-7.36 (m, 1H, 9-H), 7.33 (m, 1H, 10-H), 4.27-4.18 (m, 2H, CONCH_2_), 3.65 (t, *J* = 4.4 Hz, 4H, *CH_2_*O*CH_2_*), 2.53 (m, 6H, 2×^+^NCH_2_, CH_2_N^+^), 2.03-1.91 (m, 2H, NCH_2_*CH_2_*CH_2_N).

N-(3-(piperazin-1-yl) propyl) benzo[*k*, *l*] selenoxanthene-3,4-dicarboximide **7a**:

R_f_: 0.19 (DCM/methanol 20/1, v/v), orange yellow solid, yield: 92%, mp: 245‒247 °C, 97.0% purity by HPLC. ^1^H NMR (500 MHz, DMSO-*d_6_*): δ 8.58 (s, 1H, NH), 8.31 (d, *J* = 8.5 Hz, 1H, 5-H), 8.25 (m, 2H, 1-H, 2-H), 8.05 (d, *J* = 7.8 Hz, 1H, 6-H), 7.79 (d, *J* = 7.8 Hz, 1H, 8-H), 7.66-7.59 (m, 1H, 11-H), 7.43-7.35 (m, 2H, 9-H, 10-H), 4.01 (t, *J* =7.7 Hz, 2H, CONCH_2_), 3.03 (m, 4H, *CH_2_*NH*CH_2_*), 2.56 (m, 4H, 2×^+^NCH_2_), 2.49 (m, 2H, CH_2_N^+^), 1.84-1.71 (m, 2H, NCH_2_*CH_2_*CH_2_N).

N-(3-(dimethylamino)propyl)benzo[*k*, *l*] selenoxanthene-3,4-dicarboximide hydrochloride **1** (Se**1**):

R_f_: 0.21 (DCM/methanol 15/1, v/v), orange solid, yield: 75%, mp: 270‒272°C, 98.1% purity by HPLC. ^1^H NMR (500 MHz, CDCl_3_): δ 6.90 (m, 4H, 1-H, 2-H, 5-H, 6-H), 6.75 (m, 2H, 8-H, 11-H), 6.49 (m, 2H, 9-H, 10-H), 3.38 (m, 2H, CONCH_2_), 3.11 (m, 2H, CH_2_N^+^), 2.89 (s, 6H, 2×CH_3_), 1.73 (m, 2H, NCH_2_*CH_2_*CH_2_N). ESI-Mass: calcd m/z for C_23_H_21_ClN_2_O_2_Se: 472.05; found: 437.20 [M-Cl]^+^. Anal. Calcd for C_23_H_21_ClN_2_O_2_Se: C, 58.55; H, 4.49; N, 5.94; Se, 16.73; found: C, 58.38; H, 4.50; N, 5.91; Se, 16.69.

N-(3-(diethylamino)propyl)benzo[*k*, *l*] selenoxanthene-3,4-dicarboximide hydrochloride **2** (Se**2**):

R_f_: 0.20 (DCM/methanol 15/1, v/v), orange yellow solid, yield: 75%, mp: 220‒222 °C, 95.9% purity by HPLC. ^1^H NMR (500 MHz, MeOD): δ 8.13 (d, *J* = 8.2 Hz, 1H, 5-H), 8.04-8.01 (m, 1H, 2-H), 7.97 (d, *J* = 8.4 Hz, 1H, 1-H), 7.92 (d, *J* = 7.8 Hz, 1H, 6-H)), 7.44 (t, *J* = 5.8 Hz, 1H, 8-H), 7.39 (m, 1H, 11-H), 7.34-7.29 (m, 2H, 9-H, 10-H), 4.16-4.10 (m, 2H, CONCH_2_), 3.33 (m, 6H, 2×^+^NCH_2_, CH_2_N^+^), 2.19-2.14 (m, 2H, NCH_2_*CH_2_*CH_2_N), 1.37 (t, *J* = 7.3 Hz, 6H, 2×CH_3_). ESI-Mass: calcd m/z for C_25_H_25_ClN_2_O_2_Se: 500.08; found: 465.10 [M-Cl]^+^. Anal. Calcd for C_25_H_25_ClN_2_O_2_Se: C, 60.07; H, 5.04; N, 5.60; Se, 15.80; found: C, 60.01; H, 5.07; N, 5.65; Se, 15.75.

N-(3-(Diethanolamino)propyl)benzo[*k*, *l*] selenoxanthene-3,4-dicarboximide hydrochloride **3** (Se**3**):

R_f_: 0.18 (DCM/methanol 12/1, v/v), yellow solid, yield: 75%, mp: 198‒200 °C, 98.6% purity by HPLC. ^1^H NMR (500 MHz, MeOD): δ 8.06 (d, *J* = 8.1 Hz, 1H, 5-H), 7.96 (d, *J* = 7.8 Hz, 1H, 2-H), 7.91-7.82 (m, 2H, 1-H, 6-H), 7.39-7.32 (m, 2H, 8-H, 11-H), 7.28 (m, 2H, 9-H, 10-H), 4.11 (t, *J* = 6.6 Hz, 2H, CONCH_2_), 4.02-3.93 (m, 4H, 2×*CH_2_*OH), 3.53-3.43 (m, 6H, 2×^+^NCH_2_, CH_2_N^+^), 2.29-2.19 (m, 2H, NCH_2_*CH_2_*CH_2_N). ESI-Mass: calcd m/z for C_25_H_25_ClN_2_O_4_Se: 532.07; found: 497.10 [M-Cl]^+^, 519.1 [M-Cl+Na]^+^. Anal. Calcd for C_25_H_25_ClN_2_O_4_Se: C, 56.45; H, 4.74; N, 5.27; Se, 14.85; found: C, 56.62; H, 4.77; N, 5.21; Se, 14.90.

N-(3-(pyrrolidin-1-yl)propyl)benzo[*k*, *l*] selenoxanthene-3,4-dicarboximide hydrochloride **4** (Se**4**):

R_f_: 0.19 (DCM/methanol 15/1, v/v), yellow solid, yield: 75%, mp: 219‒221 °C, 97.1% purity by HPLC. ^1^H NMR (500 MHz, D_2_O): δ 6.91 (m, 8H, 1-H, 2-H, 5-H, 6-H, 8-H, 10-H, 9-H, 11-H), 3.74-2.69 (m, 8H, CONCH_2_, 2×^+^NCH_2_, CH_2_N^+^), 2.21-1.49 (m, 6H, NCH_2_*CH_2_*CH_2_N, 2×CH_2_ (cyclo)). ESI-Mass: calcd m/z for C_25_H_23_ClN_2_O_2_Se: 498.06; found: 463.10 [M-Cl]^+^. Anal. Calcd for C_25_H_23_ClN_2_O_2_Se: C, 60.31; H, 4.66; N, 5.63; Se, 15.86; found: C, 60.16; H, 4.62; N, 5.68; Se, 15.79.

N-(3-(piperidin-1-yl)propyl)benzo[*k*, *l*] selenoxanthene-3,4-dicarboximide hydrochloride **5** (Se**5**):

R_f_: 0.22 (DCM/methanol 20/1, v/v), pale yellow solid, yield: 75%, mp: 231‒233 °C, 97.6% purity by HPLC. ^1^H NMR (500 MHz, MeOD): δ 8.37-7.66 (m, 4H, 1-H, 2-H, 5-H, 6-H), 7.30 (m, 4H, 8-H, 9-H, 10-H, 11-H), 4.10 (m, 2H, CONCH_2_), 3.64 (m, 2H, ^+^NCH_2_), 3.26-3.03 (m, 4H, ^+^NCH_2_, CH_2_N^+^), 2.20 (m, 2H, NCH_2_*CH_2_*CH_2_N), 2.07-1.49 (m, 6H, 3 × CH_2_ (cyclo)). ESI-Mass: calcd m/z for C_26_H_25_ClN_2_O_2_Se: 512.08; found: 477.10 [M-Cl]^+^. Anal. Calcd for C_26_H_25_ClN_2_O_2_Se: C, 61.00; H, 4.92; N, 5.47; Se, 15.42; found: C, 61.26; H, 4.90; N, 5.50; Se, 15.37.

N-(3-morpholinopropyl)benzo[*k*, *l*] selenoxanthene-3,4-dicarboximide hydrochloride **6** (Se**6**):

R_f_: 0.19 (DCM/methanol 15/1, v/v), yellow solid, yield: 75%, mp: 223‒225 °C, 96.6% purity by HPLC. ^1^H NMR (500 MHz, CDCl_3_) δ 6.97 (m, 4H, 1-H, 2-H, 5-H, 6-H), 6.79 (m, 2H, 8-H, 11-H), 6.61-6.52 (m, 2H, 9-H, 10-H), 4.00 (m, 4H, *CH_2_*O*CH_2_*), 3.46 (m, 2H, CONCH_2_), 3.29 (m, 4H, 2×^+^NCH_2_), 3.11 (m, 2H, CH_2_N^+^), 1.78 (m, 2H, NCH_2_*CH_2_*CH_2_N). ESI-Mass: calcd m/z for C_25_H_23_ClN_2_O_3_Se: 514.06; found: 479.10 [M-Cl]^+^. Anal. Calcd for C_25_H_23_ClN_2_O_3_Se: C, 58.43; H, 4.51; N, 5.45; Se, 15.37; found: C, 58.56; H, 4.54; N, 5.42; Se, 15.44.

N-(3-(piperazin-1-yl) propyl) benzo[*k*, *l*] selenoxanthene-3,4-dicarboximide hydrochloride **7** (Se**7**):

R_f_: 0.18 (DCM/methanol 15/1, v/v), orange yellow solid, yield: 75%, mp: 247‒249 °C, 96.3% purity by HPLC. ^1^H NMR (500 MHz, CDCl_3_): δ 7.02 (m, 4H, 1-H, 2-H, 5-H, 6-H), 6.86-6.76 (m, 2H, 8-H, 11-H), 6.61 (m, 2H, 9-H, 10-H), 3.71 (m, 8H, 4×^+^NCH_2_), 3.52 (m, 2H, CONCH_2_), 3.33 (m, 2H, CH_2_N^+^), 1.88 (m, 2H, NCH_2_*CH_2_*CH_2_N). ESI-Mass: calcd m/z for C_25_H_25_Cl_2_N_3_O_2_Se: 549.05; found: 478.1 [M-2Cl-H]^+^, 522.1[M-2Cl+2Na-H]^+^. Anal. Calcd for C_25_H_25_Cl_2_N_3_O_2_Se: C, 54.66; H, 4.59; N, 7.65; Se, 14.37; found: C, 54.47; H, 4.57; N, 7.61; Se, 14.42.

**
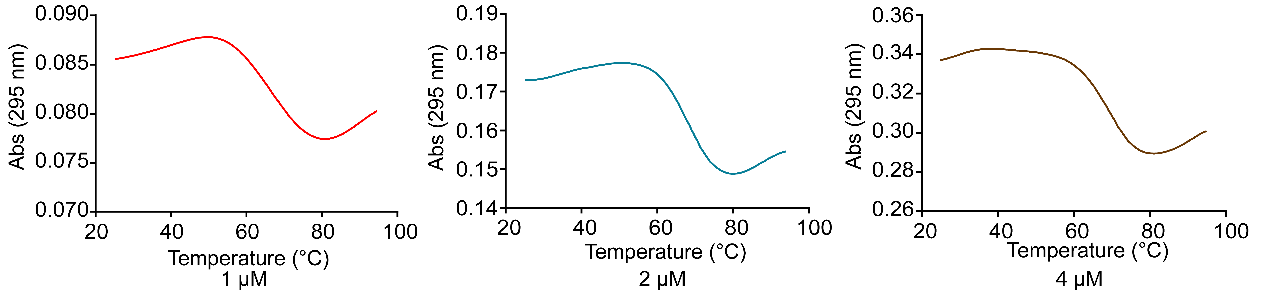
Supplementary Figures**

**Figure S1. UV-melting curves of 1, 2, 4 μM TMPRSS2-G resolved in Tris-HCl buffer (5 mM KCl).**

**
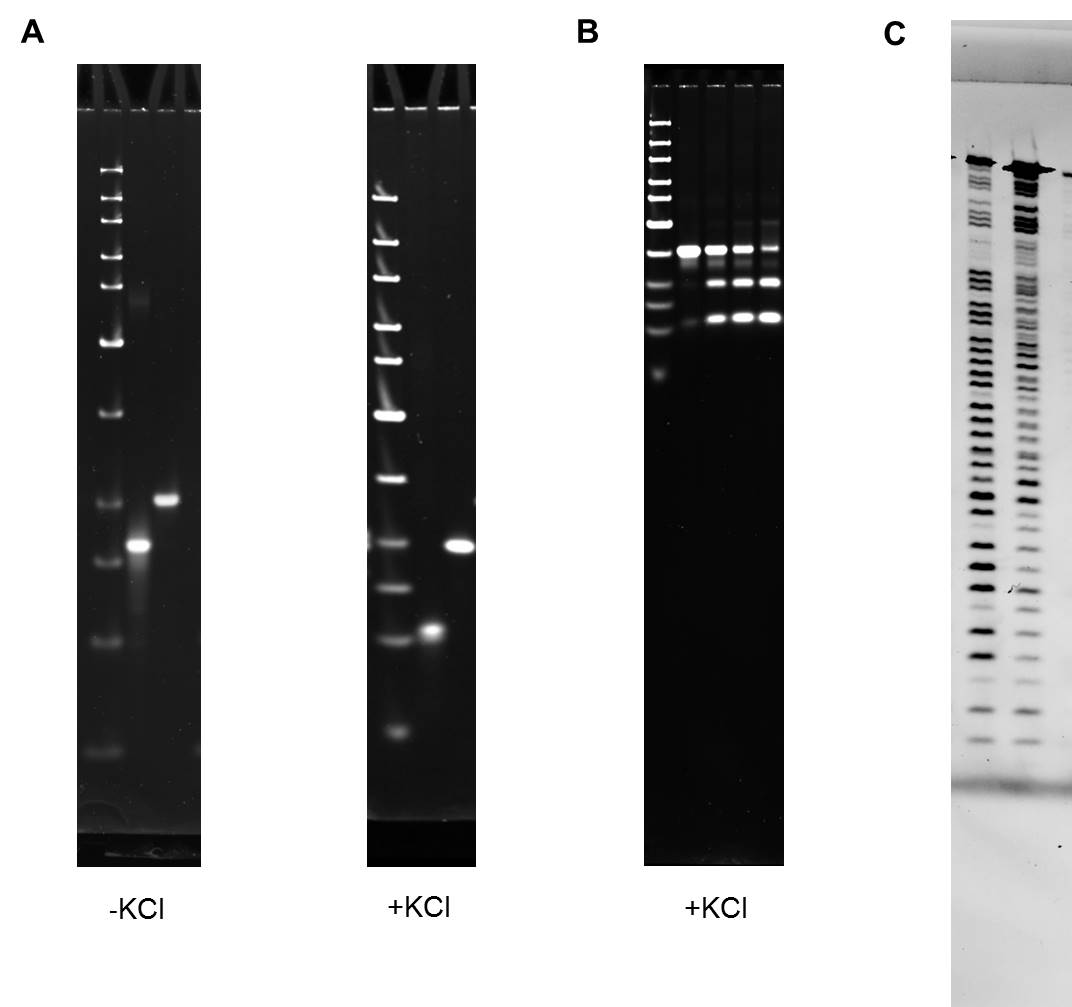
**

**Figure S2. Original images for Figures 2A (A), 2B (B) and 2F (C)** **in the manuscript text.**

**
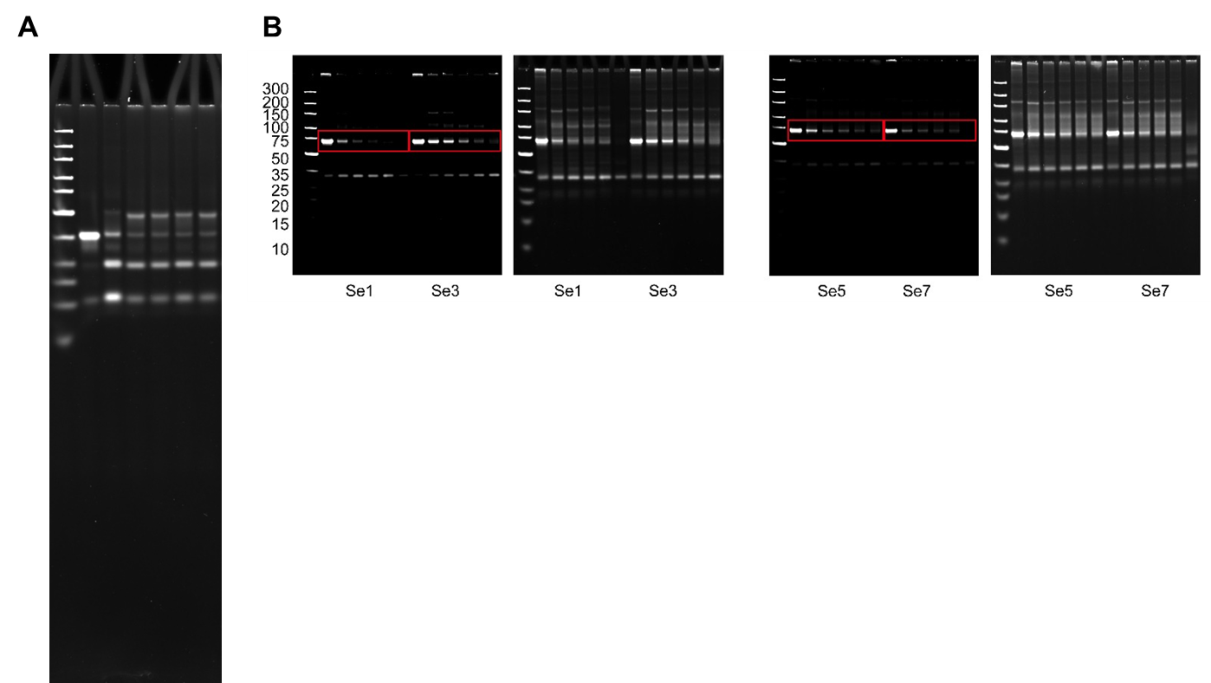
**

**Figure S3. Uncropped image for Figure 3C (A), 3D (B) in the manuscript text.** The boxed regions in Figure **S3B** are presented in the indicated figures in the manuscript text.


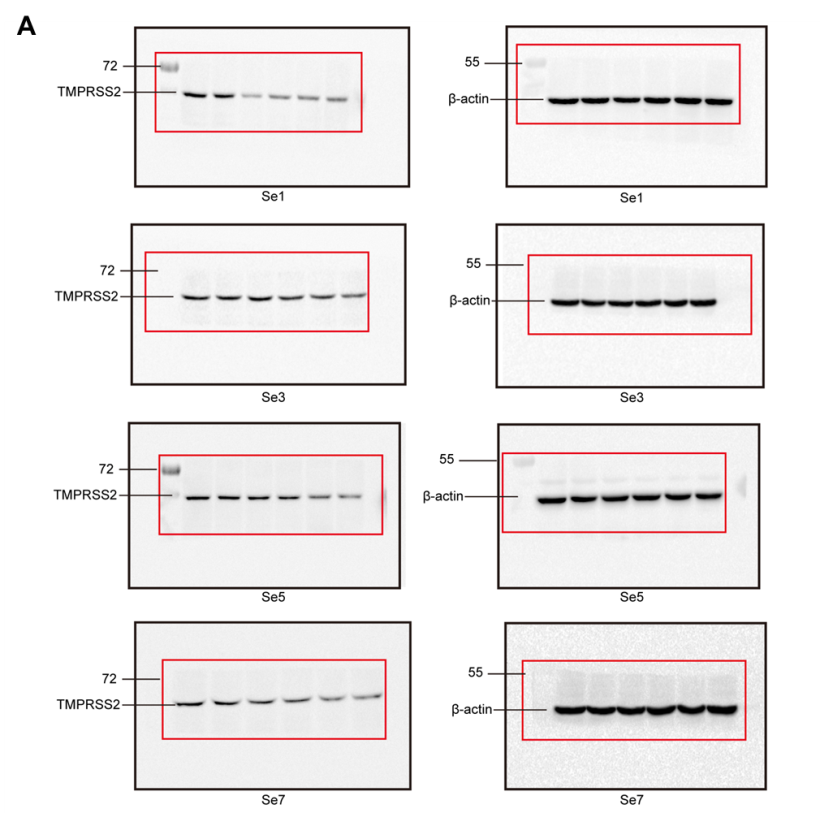


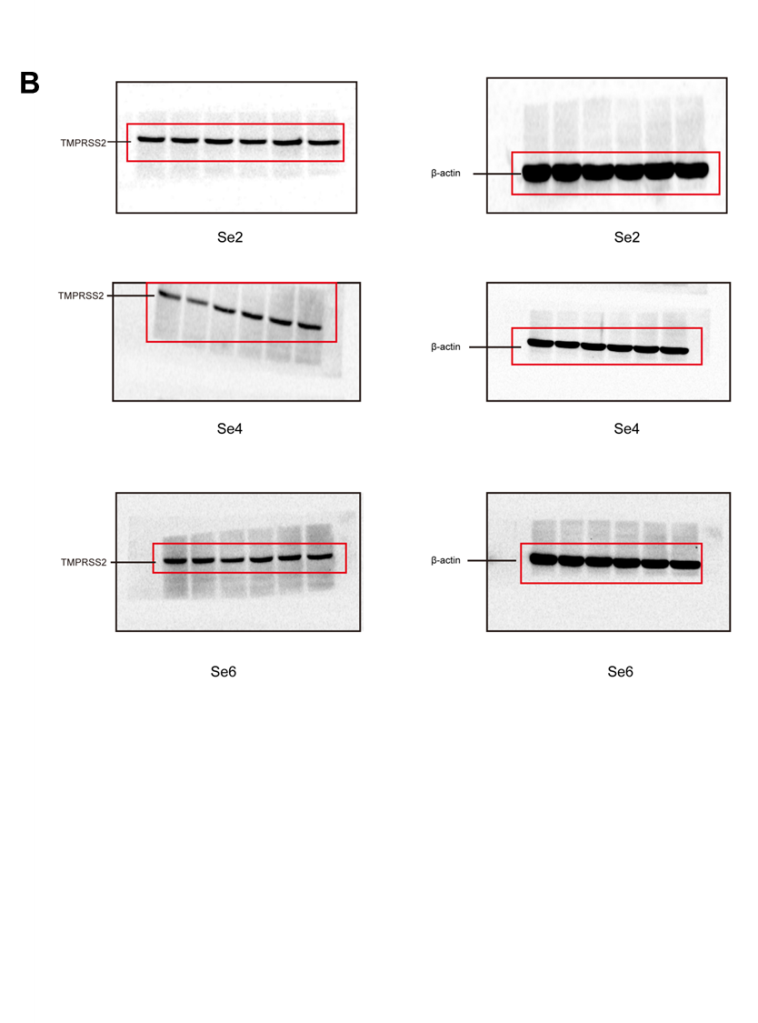


**Figure S4. Uncropped image for Figure 4B (A and B) in the manuscript text.** (**A**) Se**1**, Se**3**, Se**5** and Se**7**; (**B**) Se**2**, Se**4** and Se**6**. The boxed regions in Figures **S4A** and **S4B** are presented in the indicated figure **4B** in the manuscript text.


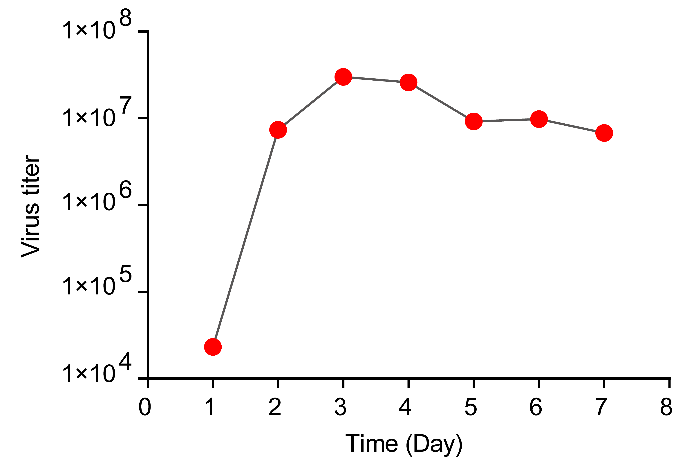


**Figure S5. Multicycle replication kinetics of influenza virus.** Calu-3 cells were infected with A/PR8/34(MOI: 0.0001), after infection, supernatants were collected every 24 h until 7 days. And viral titers were determined by plaque assay. The virus titer was presented as plaque-forming units per ml (pfu/ml).


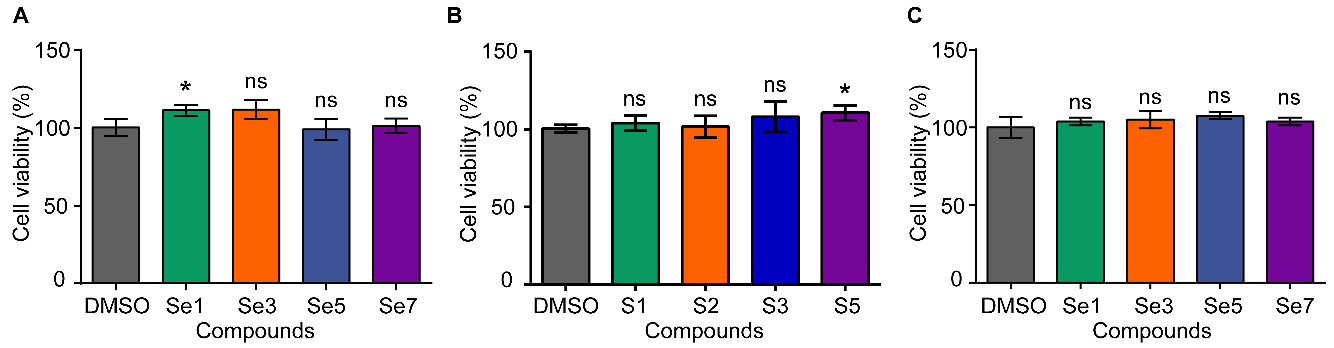


**Figure S6. Cytotoxicity of benzoselenoxanthene and benzothioxanthene analogues.** (A) Calu-3+10 μM benzoselenoxanthene derivatives. (B) Calu-3+10 μM benzothioxanthene analogues^1^. (C) A549+1 μM benzoselenoxanthene analogues.


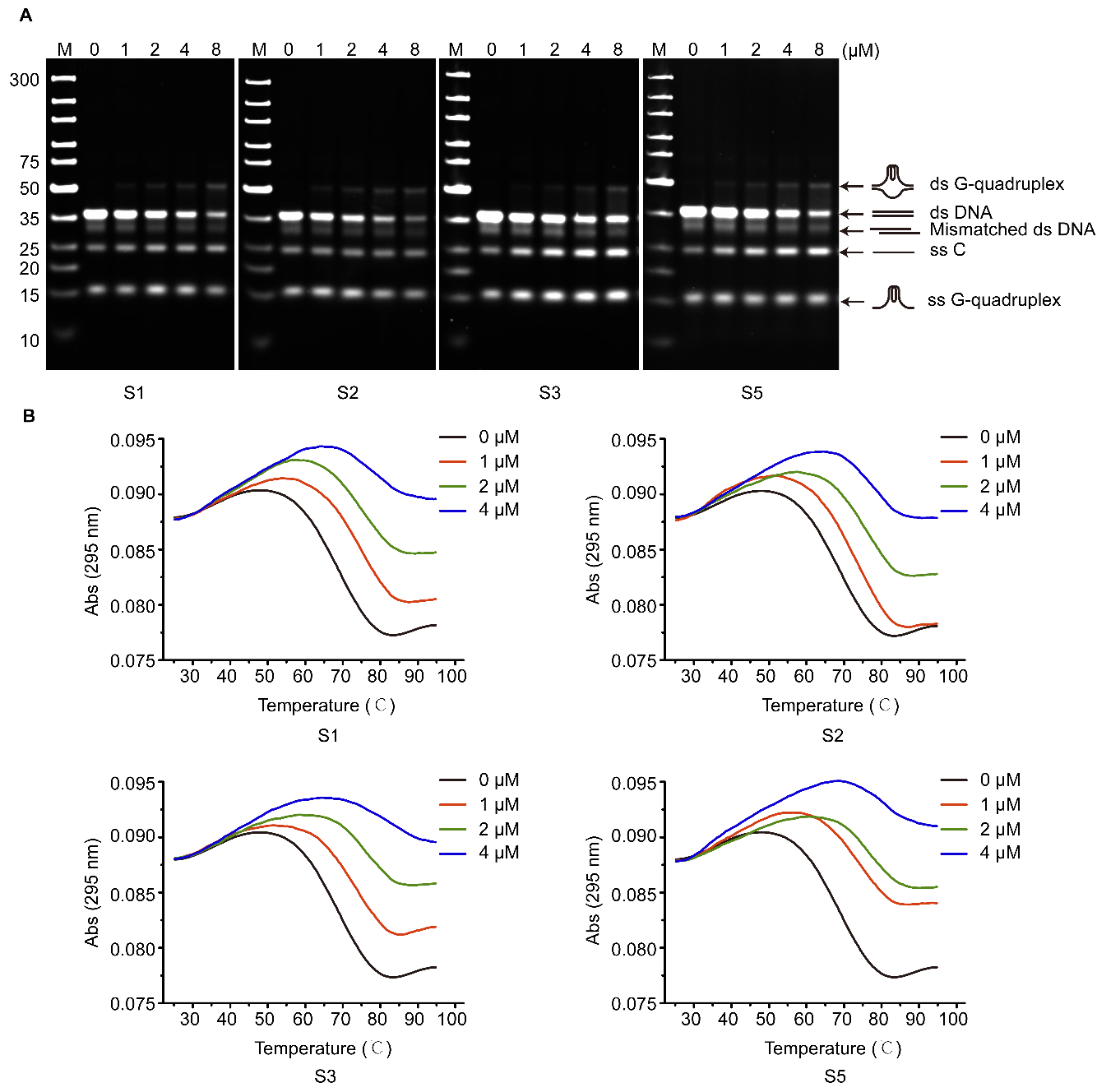


**Figure S7. Benzothioxanthene analogues**^1^ **stabilize TMPRSS2 G-quadruplex.** (A) Benzothioxanthene analogues stabilized TMPRSS2 G-quadruplex structure in the presence of complementary strand (TMPRSS-C). TMPRSS2-G and TMPRSS2-C were incubated with or without compounds, after denaturation and renaturation, samples were subjected to native PAGE contained 50 mM KCl. M: marker; ds G-quadruplex: complementary dsDNA contained G-quadruplex in the G-rich strand, ds NDA: complementary double strand DNA, mismatched dsDNA: partly complementary dsDNA, ss C: TMPRSS2-C, ss G-quadruplex: G-quadruplex formatted by TMPRSS2-G. (B) Benzothioxanthene analogues increased thermal stability of TMPRSS2 G-quadruplex. TMPRSS2-G was incubated with different concentrations of compounds in Tris-HCl buffer containing 5 mM KCl, after denaturation and renaturation, samples were subjected to UV-melting assay.


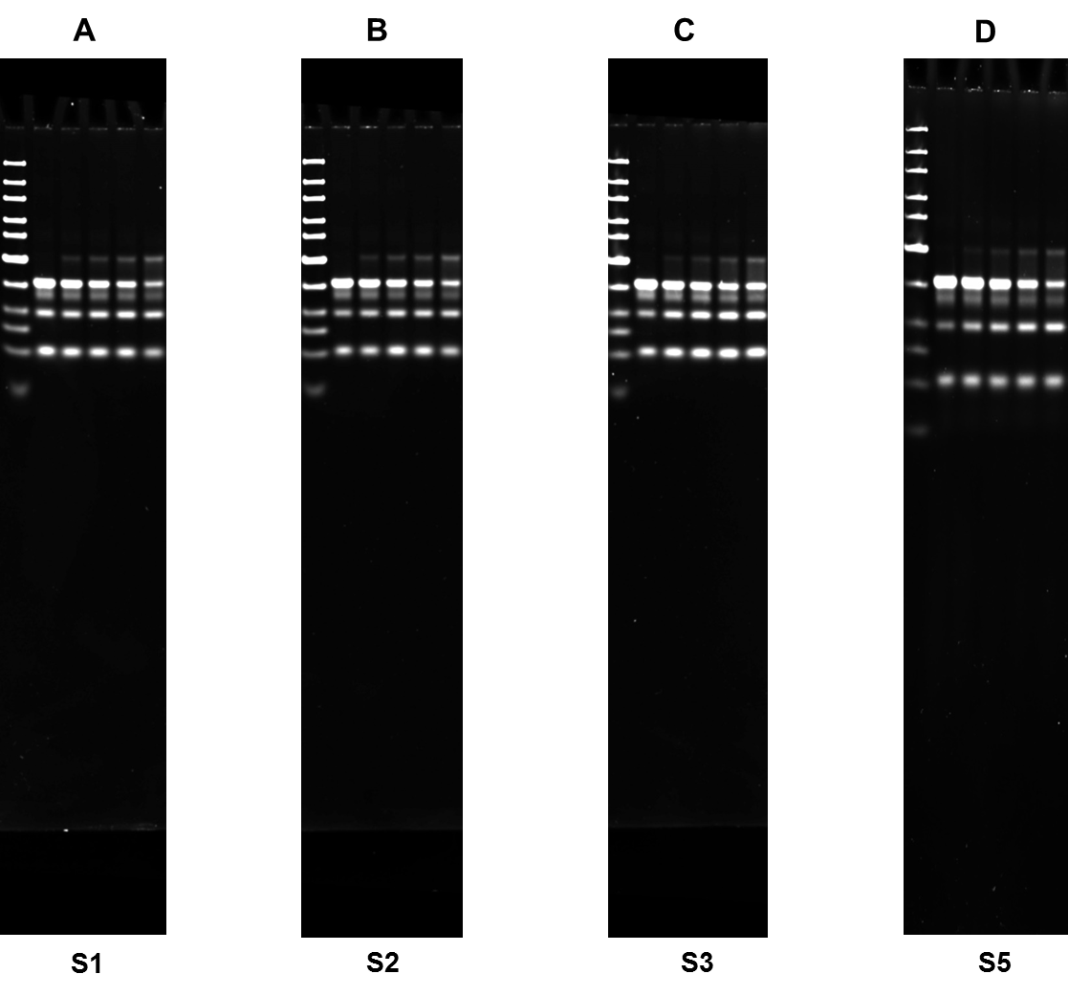


**Figure S8. Original images for Figure S7A in the Supplementary information.** (A) Benzothioxanthene analogue **S1**^1^; (B) Benzothioxanthene analogue **S2**^1^; (C) Benzothioxanthene analogue **S3**^1^; (D) Benzothioxanthene analogue **S5**^1^***.***


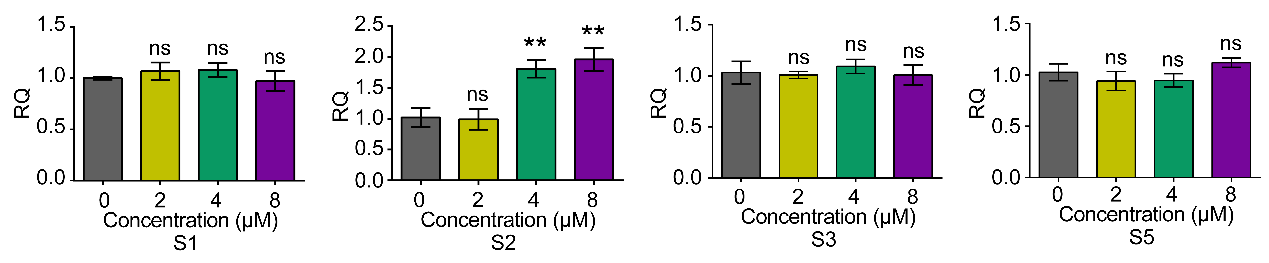


**Figure S9. Benzothioxanthene analogues**^1^ **cannot down-regulate TMPRSS2 gene transcription.**

**Supplementary tables**

**Table S1.** Sequences of oligomers/primers used in this study

| **Assay** | **Oligonucleotide** | **Sequence** |
| --- | --- | --- |
| Competition EMSA | Sp1-Biotin | 5′-Biotin-GGGCGGGGGCGGGGGCGGCGG-Biotin-3′ |
|  | Sp1-Cold | 5′-GGGCGGGGGCGGGGGCGGCGG-3′ |
|  | Sp1-Mut | 5′-GTGCGGTAGCGGGTGCGGCGG-3′ |
|  | EGR1-Biotin | 5′-Biotin-GGGCGGGCGGGGGCGGGGGCGGCGG-Biotin-3′ |
|  | EGR1-Cold | 5′-GGGCGGGCGGGGGCGGGGGCGGCGG-3′ |
|  | EGR1-Mut | 5′-GGGCGTGCGGTAGCGGGTGCGGCGG-3′ |
| EMSA/CD/UV melting assay | TMPRSS2-G | 5′-CCTGGGCGGGCGGGGGCGGGGGCGGCGGGAGGAGG-3′ |
|  | TMPRSS2-C | 5′-CCTCCTCCCGCCGCCCCCGCCCCCGCCCGCCCAGG­­­-3′ |
|  | TMPRSS2-G-Mut | 5′-CCTGTGCGTGCGTGTGCGTGTGCGTCGTGATGAGT-3′ |
|  | TMPRSS2-C-Mut | 5′-ACTCATCACGACGCACAACGCACACGCACGCACAGG-3′ |
| DMS footprinting | TMPRSS2-G-FAM | 5′-AGCGGGTCGCGGGTCACCCTGGGCGGGCGG  GGGCGGGGGCGGCGGGAGGAGGCGTTTCC-FAM-3′ |
| PCR Stop Assay | TM-up | 5′-TCACCCTGGGCGGGCGGGGG  CGGGGGCGGCGGGAGGAGG-3′ |
|  | TM-rev | 5′-GCCGCCCTCCTCCTAAGTATC  TAACTAGCTACTGCTTAGCTA-3′ |
| RT-PCR | P-TMPRSS2-F | 5′-CTGGTGGCTGATAGGGGAT-3′ |
|  | P-TMPRSS2-R | 5′-GTCTGCCCTCATTTGTCGAT-3′ |
|  | P-GAPDH-F | 5′-AGAAGGCTGGGGCTCATTTG-3′ |
|  | P-GAPDH-R | 5′-AGGGGCCATCCACAGTCTTC-3′ |

**Table S2.** Summary of wild/mutant promoter transfection data analysis (for Figure 1B).

| **Table Analyzed** | **Pro-wild/mut** |
| --- | --- |
|  | Pro-mut |
| vs | vs |
|  | Pro-wild |
| P value | ＜0.0001 |
| P value summary | *** |
| t, df | t=29.82 df=4 |

**Table S3.** Summary of Wild/Mut promoter and pCMV-Sp1/EGR1co-transfection data analysis (for Figure 1D).

| **TF** | **Sp1** | | **EGR1** | |
| --- | --- | --- | --- | --- |
|  | Wild | Mut | Wild | Mut |
| vs | vs | vs | Vs | vs |
|  | Wild +Sp1 | Mut+Sp1 | Wild+EGR1 | Mut+EGR1 |
| P value | < 0.0001 | 0.0007 | 0.0001 | 0.8864 |
| P value summary | *** | *** | *** | ns |
| t, df | t=99.38 df=4 | t=9.423 df=4 | t=15.16 df=4 | t=0.1523 df=4 |

**Table S4.** Δ*T_m_* (°C) values for TMPRSS2-G interaction with derivatives (for Figure 3B).

| **Compounds** | **Se1** | **Se3** | **Se5** | **Se7** |
| --- | --- | --- | --- | --- |
| 2 μM | 8.50 | 3.75 | 8.75 | 8.25 |
| 4 μM | 14.25 | 7.25 | 13.75 | 14.25 |
| 8 μM | 19.75 | 10.25 | 20.25 | 19.25 |

**Table S5.** Summary of RT-PCR data analysis (for Figure 4A).

| **Compounds** | **Se1** | **Se1** | **Se1** | **Se3** | **Se3** | **Se3** |
| --- | --- | --- | --- | --- | --- | --- |
|  | 2 μM | 4 μM | 8 μM | 2 μM | 4 μM | 8 μM |
| vs | vs | vs | vs | vs | vs | vs |
|  | 0 μM | 0 μM | 0 μM | 0 μM | 0 μM | 0 μM |
| P value | 0.0476 | 0.0134 | 0.0043 | 0.0109 | 0.0006 | 0.0001 |
| P value summary | * | * | ** | * | *** | *** |
| t, df | t=2.824 df=4 | t=4.232 df=4 | t=5.827 df=4 | t=4.486 df=4 | t=9.757 df=4 | t=14.73 df=4 |
| **Compounds** | **Se5** | **Se5** | **Se5** | **Se7** | **Se7** | **Se7** |
|  | 2 μM | 4 μM | 8 μM | 2 μM | 4 μM | 8 μM |
| vs | vs | vs | vs | vs | vs | vs |
|  | 0 μM | 0 μM | 0 μM | 0 μM | 0 μM | 0 μM |
| P value | 0.038 | 0.0076 | 0.0007 | 0.0091 | 0.0047 | 0.0023 |
| P value summary | * | ** | *** | ** | ** | ** |
| t, df | t=3.050 df=4 | t=4.979 df=4 | t=9.286 df=4 | t=4.724 df=4 | t=5.688 df=4 | t=6.913 df=4 |

**Table S6.** Summary of wild promoter transfection with derivatives data analysis (for Figur 4C).

| **Promoter-wild** | **Se1** | **Se3** | | **Se5** | | **Se7** |  |
| --- | --- | --- | --- | --- | --- | --- | --- |
|  | Se1 | Se3 | | Se5 | | Se7 |  |
| vs | vs | vs | | Vs | | vs |  |
|  | DMSO | DMSO | | DMSO | | DMSO |  |
| P value | 0.0001 | 0.0003 | | 0.0063 | | 0.0002 |  |
| P value summary | *** | *** | | ** | | *** |  |
| t, df | t=14.53 df=4 | t=11.33 df=4 | | t=5.248 df=4 | | t=13.07 df=4 |  |
| **Promoter-Mut** | **Se1** | | **Se3** | | **Se5** | **Se7** | |
|  | Se1 | | Se3 | | Se5 | Se7 | |
| vs | vs | | vs | | Vs | vs | |
|  | DMSO | | DMSO | | DMSO | DMSO | |
| P value | 0.0255 | | 0.0590 | | 0.2298 | 0.0398 | |
| P value summary | * | | ns | | Ns | * | |
| t, df | t=3.475 df=4 | | t=2.617 df=4 | | t=1.416 df=4 | t=3.003 df=4 | |

**Table S7.** Summary of antiviral activity data analysis (for Figure 5A).

| **Compounds** | | |  | **Se1** | | **Se1** | | **Se1** | | **Se3** | | **Se3** | | **Se3** | |
| --- | --- | --- | --- | --- | --- | --- | --- | --- | --- | --- | --- | --- | --- | --- | --- |
|  | | |  | 2 μM | | 4 μM | | 8 μM | | 2 μM | | 4 μM | | 8 μM | |
| vs | | |  | vs | | vs | | vs | | vs | | vs | | vs | |
|  | | |  | 0 μM | | 0 μM | | 0 μM | | 0 μM | | 0 μM | | 0 μM | |
| P value | | |  | 0.1682 | | 0.001 | | 0.0003 | | 0.1301 | | 0.0027 | | 0.0012 | |
| P value summary | | |  | ns | | *** | | *** | | ns | | ** | | ** | |
| t, df | | |  | t=1.680 df=4 | | t=8.698 df=4 | | t=11.85 df=4 | | t=1.901 df=4 | | t=6.604 df=4 | | t=8.165 df=4 | |
| **Compounds** | | |  | **Se5** | | **Se5** | | **Se5** | | **Se7** | | **Se7** | | **Se7** | |
|  | | |  | 2 μM | | 4 μM | | 8 μM | | 2 μM | | 4 μM | | 8 μM | |
| vs | | |  | vs | | vs | | vs | | vs | | vs | | vs | |
|  | | |  | 0 μM | | 0 μM | | 0 μM | | 0 μM | | 0 μM | | 0 μM | |
| P value | | |  | 0.0221 | | 0.0038 | | 0.0007 | | 0.0035 | | 0.0022 | | 0.0001 | |
| P value summary | | |  | * | | ** | | *** | | ** | | ** | | *** | |
| t, df | |  | t=3.635 df=4 | | t=6.046 df=4 | | t=9.551 df=4 | | t=6.161 df=4 | | t=7.017 df=4 | | t=15.21 df=4 | |  |
| **Compounds** | | |  | **oseltamivir** | | **oseltamivir** | | **oseltamivir** | | **camostat** | | **camostat** | | **camostat** | |
|  | | |  | 2 μM | | 4 μM | | 8 μM | | 2 μM | | 4 μM | | 8 μM | |
| vs | | |  | vs | | vs | | vs | | vs | | vs | | vs | |
|  | | |  | 0 μM | | 0 μM | | 0 μM | | 0 μM | | 0 μM | | 0 μM | |
| P value | | |  | 0.0519 | | 0.0135 | | 0.0004 | | 0.002 | | 0.0006 | | 0.0006 | |
| P value summary | | |  | ns | | * | | *** | | ** | | *** | | *** | |
| t, df | | |  | t=2.741 df=4 | | t=4.221 df=4 | | t=10.89 df=4 | | t=7.192 df=4 | | t=9.816 df=4 | | t=9.843 df=4 | |

**Table S8.** Summary of data analysis (for Figure 5C)

| **Compounds** | **Se1** | **Se3** | **Se5** | **Se7** | **Oseltamivir** |
| --- | --- | --- | --- | --- | --- |
|  | vs | vs | vs | vs | vs |
|  | Se**1**+ trypsin | Se**3**+ trypsin | Se**5**+ trypsin | Se**7**+ trypsin | Oseltamivir+ trypsin |
| P value | 0.0020 | 0.0002 | 0.0466 | 0.0441 | 0.3713 |
| P value summary | ** | *** | * | * | ns |
| t, df | t=7.201 df=4 | t=12.99 df=4 | t=2.845 df=4 | t=2.901 df=4 | t=1.006 df=4 |
| **Compounds** | **DMSO** | **DMSO** | **DMSO** | **DMSO** | **DMSO** |
|  | vs | vs | vs | vs | vs |
|  | Se**1**+ trypsin | Se**3**+ trypsin | Se**5**+ trypsin | Se**7**+ trypsin | Oseltamivir+ trypsin |
| P value | 0.1310 | 0.5790 | 0.0651 | 0.0249 | 0.0026 |
| P value summary | ns | ns | ns | * | ** |
| t, df | t=1.895 df=4 | t=0.6030 df=4 | t=2.524 df=4 | t=3.500 df=4 | t=6.668 df=4 |

**Table S9.** Summary of cells cytotoxicity data analysis (for Figure S4).

| **Calu-3/Compounds** | **Se1** | **Se3** | **Se5** | **Se7** |
| --- | --- | --- | --- | --- |
|  | 8 μM | 8 μM | 8 μM | 8 μM |
| vs | vs | vs | vs | vs |
|  | 0 μM | 0 μM | 0 μM | 0 μM |
| P value | 0.0444 | 0.0754 | 0.8076 | 0.8212 |
| P value summary | * | ns | ns | ns |
| t, df | t=2.894 df=4 | t=2.387 df=4 | t=0.2601 df=4 | t=0.2413 df=4 |
| **Calu-3/Compounds** | **S1** | **S2** | **S3** | **S5** |
|  | 8 μM | 8 μM | 8 μM | 8 μM |
| vs | vs | vs | vs | vs |
|  | 0 μM | 0 μM | 0 μM | 0 μM |
| P value | 0.1594 | 0.4374 | 0.1722 | 0.0412 |
| P value summary | * | ns | ns | * |
| t, df | t=1.726 df=4 | t=0.8617 df=4 | t=1.660 df=4 | t=2.968 df=4 |
| **A549/Compounds** | **Se1** | **Se3** | **Se5** | **Se7** |
|  | 1 μM | 1 μM | 1μM | 1 μM |
| vs | vs | vs | vs | vs |
|  | 0 μM | 0 μM | 0 μM | 0 μM |
| P value | 0.4023 | 0.3714 | 0.136 | 0.4023 |
| P value summary | ns | ns | ns | ns |
| t, df | t=0.9359 df=4 | t=1.006 df=4 | t=1.863 df=4 | t=0.9359 df=4 |

**Table S10.** Summary of RT-PCR data analysis (for Figure S6)

| **Compounds** | **S1** | **S1** | **S1** | **S2** | **S2** | **S2** |
| --- | --- | --- | --- | --- | --- | --- |
|  | 2 μM | 4 μM | 8 μM | 2 μM | 4 μM | 8 μM |
| vs | vs | vs | vs | vs | vs | vs |
|  | 0 μM | 0 μM | 0 μM | 0 μM | 0 μM | 0 μM |
| P value | 0.249 | 0.1201 | 0.6397 | 0.8154 | 0.0027 | 0.0026 |
| P value summary | ns | ns | ns | ns | ** | ** |
| t, df | t=1.348 df=4 | t=1.970 df=4 | t=0.5056 df=4 | t=0.2493 df=4 | t=6.599 df=4 | t=6.695 df=4 |
| **Compounds** | **S3** | **S3** | **S3** | **S5** | **S5** | **S5** |
|  | 2 μM | 4 μM | 8 μM | 2 μM | 4 μM | 8 μM |
| vs | vs | vs | vs | vs | vs | vs |
|  | 0 μM | 0 μM | 0 μM | 0 μM | 0 μM | 0 μM |
| P value | 0.7657 | 0.4608 | 0.7993 | 0.3019 | 0.2663 | 0.1597 |
| P value summary | ns | ns | ns | ns | ns | ns |
| t, df | t=0.3190 df=4 | t=0.8150 df=4 | t=0.2717 df=4 | t=4.724 df=4 | t=5.688 df=4 | t=6.913 df=4 |

**Reference**

1. Zhang, W. et al. Formation and stabilization of the telomeric antiparallel G-quadruplex and inhibition of telomerase by novel benzothioxanthene derivatives with anti-tumor activity. *Sci Rep-Uk* **5**, 13693 (2015)
